# Characterization and Analysis of the Temporal and Spatial Dynamic of Several Enteritis Modeling Methodologies

**DOI:** 10.3389/fimmu.2021.727664

**Published:** 2021-12-22

**Authors:** Huangru Xu, Fangfang Cai, Ping Li, Xiaoyang Wang, Yingying Yao, Xiaoyao Chang, Zhiqian Bi, Huisong Sun, Hongqin Zhuang, Zi-Chun Hua

**Affiliations:** ^1^ The State Key Laboratory of Pharmaceutical Biotechnology, College of Life Sciences, Nanjing University, Nanjing, China; ^2^ School of Biopharmacy, China Pharmaceutical University, Nanjing, China; ^3^ Changzhou High-Tech Research Institute of Nanjing University, Changzhou, China; ^4^ Jiangsu TargetPharma Laboratories Inc., Changzhou, China

**Keywords:** enteritis, DSS, anti-CD3 antibody, HFD, inflammatory cytokines

## Abstract

Inflammatory bowel disease (IBD), such as Crohn’s disease and ulcerative colitis, is a complex disease involving genetic, immune, and microbiological factors. A variety of animal models of IBD have been developed to study the pathogenesis of human IBD, but there is no model that can fully represent the complexity of IBD. In this study, we established two acute enteritis models by oral 3% DSS or intraperitoneal injection of anti-CD3 antibody, and two chronic enteritis models by feeding 3 cycles of 1.5% DSS or 3 months of the high-fat diet, respectively, and then examined the clinical parameters, histological changes, and cytokine expression profiles after the successful establishment of the models. Our results indicated that in 3% DSS-induced acute enteritis, the colorectal injury was significantly higher than that of the small intestine, while in anti-CD3 antibody-induced acute enteritis, the small intestine injury was significantly higher than that of colorectal damage. Besides, in the 1.5% DSS-induced chronic enteritis, the damage was mainly concentrated in the colorectal, while the damage caused by long-term HFD-induced chronic enteritis was more focused on the small intestine. Therefore, our work provides a reference for selecting appropriate models when conducting research on factors related to the pathogenesis of IBD or evaluating the potential diagnosis and treatment possibilities of pharmaceuticals.

## Introduction

Inflammatory bowel disease (IBD) mainly includes Crohn’s disease (CD) and ulcerative colitis (UC). It is a complex multifactorial disease of the gastrointestinal tract caused by genetic and environmental factors (1, 2). IBD is characterized by diarrhea, bloody stools, and weight loss. Its histological features include crypt abscesses, distortion and loss, ulcerative ulcers, and infiltration of large numbers of immune cells (namely, neutrophils, monocytes, and lymphocytes). In the past few decades, many different models of experimental IBD have been developed to investigate pathogenesis to improve treatment options, which are generally divided into five categories: inducible colitis models (3–5), gene knockout (KO) models (6–10), transgenic models (11–13), spontaneous colitis models (14, 15), and adoptive transfer models (16). Although no single model can fully represent the complexity of IBD, various models provide us with indispensable tools for studying the pathogenic characteristics of IBD, identifying new genes, novel immunological molecules, and processes that may be related to the pathogenesis of IBD.

Most commonly, experimental enteritis is induced by dextran sodium sulfate (DSS) because of its simplicity and controllability in duration and severity (17). DSS firstly interfered with intestinal barrier function: mucin and goblet cell depletion, epithelial erosion, ulceration, then granulocytes infiltrate the lamina propria and submucosa, leading to the immune response. Therefore, DSS is commonly used to induce the mouse model of acute colitis that can simulate clinical and histological features of IBD with UC characteristics (18–20).

Emphasizing the role of intestinal barrier dysfunction in disease pathogenesis, we also established an *in vivo* model of acute immune-mediated diarrheal disease. Systemic T cell activation induced by administration of anti-CD3 antibodies causes acute diarrhea in humans and mice (21, 22), which is related to intestinal barrier dysfunction (23). As an effective way to induce the acute, self-limited, cytokine-mediated diarrhea in mice, the small intestinal serosa of anti-CD3 antibody-treated mice showed classic signs of inflammation, namely, vascular injection and edema (23, 24).

DSS is often used to generate chronic enteritis models by changing the concentration and cycles of administration (5, 25). In the chronic phase of DSS-induced colitis, dysplasia that resembles the clinical course of human UC frequently occurs, such as crypt architecture disarray and widening gap between the base of crypt and muscular, accompanied by increased deep mucosal lymphocytes (19). Furthermore, transepithelial migration of neutrophils leading to cryptitis and crypt abscess, a common histologic finding in human IBD, may occur even in mice subjected to the chronic DSS administration (19).

In addition to DSS, studies have found that a long-term high-fat diet (HFD) can also induce low-grade chronic intestinal inflammation in mice (26–28). Epidemiological studies have implicated that high-fat diet intake may result in an increased risk of IBD (29). HFD feeding can cause spontaneous goblet cell dysfunction, impaired mucosal barrier function, and inflammation (30).

Although these enteritis models’ clinical and histological parameters have been well established, the temporal and spatial dynamic spectrums of cytokine profile and their correlation with other parameters are still unclear. In this work, a rigorous analysis of the general profile of acute enteritis induced by DSS and anti-CD3 antibodies was performed, namely, the clinical and histological characteristics, and the temporal and spatial distribution of inflammatory mediators. In addition, we also conducted clinical, histological, and inflammatory mediators comparison studies on chronic enteritis induced by long-term DSS or HFD feeding.

## Materials and Methods

### Animal Models

C57BL/6 mice were purchased from the Beijing Animal Centre (Beijing, China) and maintained under specific pathogen-free (SPF) conditions for 1 week before the experiments. All animal experiments were approved by the Nanjing University Animal Care and Use Committee and were carried out in strict accordance with the guidelines and recommendations of the Animal Protection Committee of Nanjing University.

### Dextran Sodium Sulfate (DSS)-Induced Acute Enteritis Model

**Figure 1:**
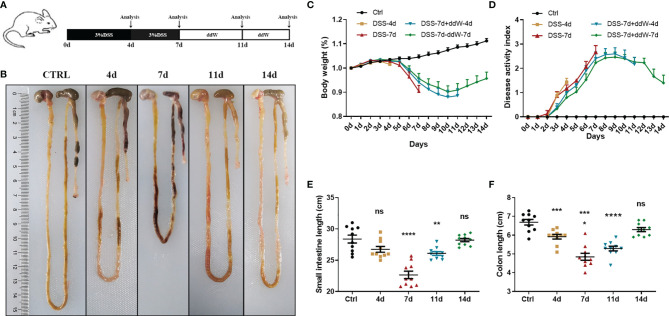
Observations of DSS-induced acute enteritis. **(A)** Mice were fed with sterile water containing 3% DSS for 7 days and then changed to sterile water for another 7 days. Mice were euthanized on the 4th, 7th, 11th, and 14th days, respectively. **(B)** Representative schematic of the whole intestine in mice sacrificed on different days (4, 7, 11, and 14 d represent DSS-4 d, DSS-7 d, DSS-7 d + ddW-4 d and DSS-7 d + ddW-7 d respectively). **(C)** Body weights of mice in each group were recorded daily and the data were shown in the percentage of the original body weight. **(D)** Disease activity index (DAI) scores during the 14 days of DSS administration (3%), which is the average of weight loss, diarrhea and rectal bleeding. **(E)** Small intestine lengths from stomach to cecum. **(F)** Colon lengths from cecum to anus. Data were represented as mean ± SEM. n = 10 mice in each group. *P < 0.05, **P < 0.01, ***P < 0.001, ****P < 0.0001. ns, no significance.

The sample animals were 6–8 weeks C57BL/6 male mice which were randomly divided into 5 groups, as is shown in **Figure 1A**. The model groups were fed with sterile water containing 3.0% (w/v) DSS (dextran sulfate sodium salt, 36–50 kDa, 0216011080, MP Biomedicals) for 7 days and then changed to sterile water for additional 7 days (31). The mice were sacrificed on the 4th, 7th, 11th and 14th days for sampling, respectively. Mice given sterile water for 14 days served as a control group. The bodyweight of mice was recorded daily.

### Anti-CD3 Antibody-Induced Acute Enteritis Model

Used for this model were 6–8 weeks C57BL/6 male mice and the animals were randomly allocated into experimental groups. To generate enteritis, 12.5 μg of anti-CD3 antibody (145-2C11, Sigma-Aldrich, St. Louis, MO) in 100 μl of phosphate-buffered saline (PBS) was intraperitoneally administered to the mice (32). The mice were sacrificed on the 1, 2, 4, 8, 12, and 24 h for sampling after treatment with anti-CD3 antibody. Mice injected with 100 μl of PBS intraperitoneally served as a control group.

### Dextran Sodium Sulfate (DSS)-Induced Chronic Enteritis Model

Eight weeks C57BL/6 male mice were received 3 cycles of DSS treatment consisting of 7 days with 1.5% DSS in sterile water followed by a 14-day recovery phase with sterile water (31). Mice given sterile water served as a control group.

### High-Fat Feed (HFD)-Induced Chronic Enteritis Model

Randomly allocated into experimental groups were 6–8 weeks C57BL/6 male mice. Mice were allowed access to water *ad libitum* and fed either an NCD or an HFD (60% of kcal from fat, 20% of kcal from carbohydrate, and 20% of kcal from protein, Research Diets) for 12 weeks. Blood glucose was measured from tail capillary blood at the end of the experiment.

### Evaluation of Disease Activity

In the DSS-induced acute enteritis model, the bodyweight of mice for each group was recorded daily and stool consistency and blood in the feces were evaluated daily using the fresh stool. Disease activity index (DAI) was calculated by scoring weight loss (0: none; 1: 1–5%; 2: 5–10%; 3: 10–20%; 4: >20%), diarrhea (0: normal; 2: loose stool; 4: diarrhea) and rectal bleeding (0: negative; 2: positive; 4: Gross bleeding), based on a previous scoring system described by Murthy et al. (33). Briefly, DAI = [(weight loss score) + (diarrhea score) + (rectal bleeding score)]/3.

### Histopathological Assessment

For pathological assessment, the middle section of each intestinal tissue was fixed in paraformaldehyde for 24 h. Then, 3 μm paraffin-embedded cross sections were prepared and stained with hematoxylin and eosin (H&E). These H&E slices were evaluated by an investigator (MAR) blinded to the experimental situation and using the scoring system described by Vieira et al. (34, 35). The histopathological score was calculated by severity of inflammation (0: none, 1: slight, 2: moderate, 3: severe), depth of injury (0: none, 1: mucosal, 2: mucosal and submucosal, 3: transmural), and crypt damage (0: none, 1: basal one-third damaged, 2: basal two-thirds damaged, 3: only surface epithelium intact, 4: the entire crypt and epithelium loss of goblet cells). The score of each parameter was multiplied by a factor reflecting the percentage of tissue involvement (1: 1–25%; 2: 26–50%; 3: 51–75%; 4: 76–100%). Briefly, histological score = (inflammation + depth of lesions + destruction of crypt) × width of lesions.

### RNA Extraction and Quantitative Real-Time PCR

Total RNA of intestinal tissue samples was extracted using the Trizol reagent (Invitrogen, Carlsbad, CA, USA) according to the manufacturer’s instructions. Approximately 1 μg of RNA was used for cDNA synthesis using the reverse transcription kit (Toyobo, Kita-Ku, Osaka, Japan). QPCR was performed using the AceQ qPCR SYBR Green Master Mix (Vazyme, Nanjing, China). The PCR cycling conditions were as follows: an initial start at 95°C for 10 min, followed by 40 cycles at 95°C for 15 s and 60°C for 1 min. The primers were listed in **Supplementary File 1** and **Table S1**. Each RT-PCR reaction was repeated at least 3 times and GAPDH or Actin was used as an internal control.

### Fecal Microbiota Analysis by 16s rRNA Sequencing

Stool samples were freshly collected from the gut and immediately frozen in liquid nitrogen. Genomic DNA was isolated from 200 mg frozen fecal samples using a QIAamp DNA Stool Mini Kit (51504, QIAGEN) according to the manufacturer’s instructions. Genomic DNA was then amplified in 50 μl triplicate reactions with bacterial 16s rRNA gene (V3-V5 region)-specific primers: 338 F (5’-ACTCCTACGGGAGGCAGCAG-3’) and 806R (5’-GGACTACHVGGGTWTCTAAT-3’). Sequencing was performed by an Illumina MiSeq PE300 system (OEbiotech Co, Ltd.). Paired-end sequences were merged to give an optimal alignment (overlap length ≥10 bp, mismatch proportion ≤20%). As an added quality control measure, the software package MacQIIME (version 1.9.1) pipeline was used to filter out and discard poor-quality reads using the default settings. Sequences were further clustered into OTUs (Operational Taxonomic Units or phylotypes) at 97% of identity using QIIME and cdhit. OTUs were assigned to closest taxonomic neighbors and relative bacterial species using Seqmatch and Blastall. Relative abundance of each OTUs and other taxonomic levels (from phylum to genus) were calculated for each sample to account for different levels of sampling across multiple individuals.

### FACS Analysis

The intestinal tissue was opened longitudinally and cut into 0.5 cm small pieces, and kept in complete medium (RPMI‐1640; Lonza, Belgium) supplemented with 10% FBS (Thermo Fisher Scientific, Belgium) and 1% antibiotic–antimycotic 100× (Thermo Fisher Scientific), incubated at 37°C for 20 min, shaken vigorously for 15 s, and filtered through a 70 µm cell strainer (Greiner Bio-One, Belgium). Repeat the above operation and combine the filtrates collected twice. The remaining tissue was digested in RPMI-1640, which was supplemented with 1 mg/ml collagenase type I (Sigma, USA), 60 U/ml DNase I (Invitrogen) and 10% FCS, 220 rpm, 37°C for 60 min, and then filtered through a 70 µm cell strainer. The filtrate was centrifuged at 1,500 rpm for 5 min to collect the cell pellet and blocked in 5% BSA for 1 h for subsequent staining. Cell types were defined based on the following markers: macrophages (CD11b^+^ F4/80^+^), CD4^+^ T cells (CD3^+^CD4^+^), CD8^+^ T cells (CD3^+^CD8^+^), neutrophils (CD11b^+^Ly6G^+^) and ILC cells (CD127^+^). All the antibodies for FACS were purchased from BD Pharmingen (San Diego, CA). Fluorescence was measured with a flow cytometer (FACS Calibur; BD Biosciences) equipped with Cell Quest software (BD Biosciences, Canada).

### Statistical Analysis

Statistical analysis was conducted by GraphPad prism 8.0. All experimental data were presented as mean ± standard error of the mean (SEM). One-way analysis of variance (ANOVA) was used to analyze the differences between groups, and a two-tailed Student’s t-test was used to analyze the significance of two groups. For all the analyses, results were reported as statistically significant if *p <*0.05.

## Results

### DSS-Induced Acute Enteritis Model

#### Weight Loss, Disease Index Score and Bowel Length Shortening

We induced acute experimental enteritis in C57BL/6 mice by adding 3% DSS to the sterile water for indicated days (**Figure 1A**). With the progress of DSS treatment, mice showed a different extent of diarrhea, grosser rectal bleeding, and shortened bowel length, which suggested the presence and development of inflammation (**Figure 1B**). During the period of DSS treatment, weight loss was detected after DSS administration under the indicated days. After 4 days of DSS treatment, the mice lose weight, which was more pronounced after 7 days. The bodyweight of mice began to rise after DSS was withdrawn for 4 days (day 11), and partially restored 7 days after DSS withdrawal (day 14) (**Figure 1C**). The disease index score (DAI) was consistent with the trend of weight change. In the 4-, and 7-day groups, DAI significantly increased with the DSS-treatment progressing compared with the normal drinking water-treated mice group. After DSS withdrawal, the symptoms gradually recovered, but the DAI of model groups was still higher than that of the control group (**Figure 1D**). Small intestine length and colon length were also measured to determine the severity of enteritis. Small intestine length shortening became the most severe in 7-day DSS-treated mice compared with drinking water-treated mice with a reduction of around 20% (**Figure 1E**). Additionally, colon length shortening became significant in 4-day DSS-treated mice and also became the most severe in 7-day DSS-treated mice with a reduction in colon length of around 28% compared with colons from drinking water-treated mice (**Figure 1F**). The reduction could be recovered after DSS withdrawal in both intestines. These results indicated that DSS could cause a significant reduction in the length of the small intestine and colon during the treatment, and the impact on the colon was more pronounced.

#### Histological Characterization

**Figure 2:**
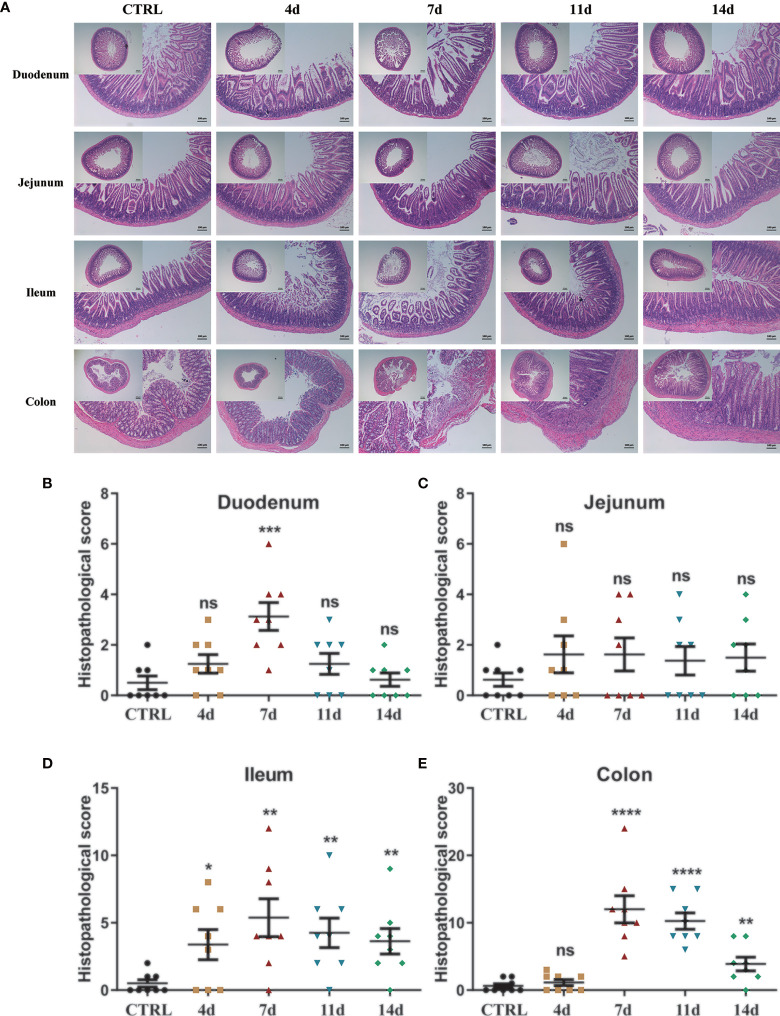
Histological observation of DSS-induced acute enteritis. **(A)** Representative paraffin sections of duodenal, jejunal, ileal and colonic tissues stained with H&E. Bars indicate 100 μm. **(B–E)** Histopathological scores of duodenal **(B)**, jejunal **(C)**, ileal **(D)**, and colonic **(E)** tissue sections (histological score = (inflammation + depth of lesions + crypt damage) × width of lesions). Data were represented as mean ± SEM. n = 8 mice in each group. *P < 0.05, **P < 0.01, ***P < 0.001, ****P < 0.0001. ns, no significance.

H&E staining further indicated the tissue morphology and inflammatory infiltration in the duodenum, jejunum, ileum, and colon, and the histopathological score was also performed. The sections of the duodenum, jejunum, ileum, and colon of the control mice all showed intact epithelium structures, no ulcers or erosions, and no inflammatory infiltration in mucosa and submucosa. Duodenum and jejunum tissues from DSS-treated mice showed no obvious inflammatory lesions. The ileum tissue of DSS-treated mice showed slight inflammatory lesions during the DSS treatment and recovered to a normal state after DSS withdrawal. However, colon tissues from DSS-treated mice showed increasingly severe inflammatory lesions extensively throughout the mucosa during the DSS treatment and attenuated after DSS withdrawal. In addition, on the 7th day of the DSS treatment group, submucosal edema increased significantly, and a lot of inflammatory cells infiltrated, which weakened slightly after DSS withdrawal (**Figure 2A**). In duodenum tissues from DSS-treated mice, the histopathological score was only increased in the 7-day group (**Figure 2B**). In jejunum tissues from DSS-treated mice, there was no significant difference in histopathological scores among groups (**Figure 2C**). Consistent with the H&E staining observation, in the ileum and colon tissues from DSS-treated mice, the histopathological score was significantly increased with the DSS treatment progressing, decreasing after DSS withdrawal (**Figures 2D–E**). The latest researches also used lipocalin-2 as an important indicator to assess the activity of intestinal inflammatory diseases (36, 37). Therefore, we also detected the lipocalin-2 mRNA levels in each intestinal segment by qPCR. Consistent with the results of H&E staining, lipocalin-2 expression levels increased significantly in the duodenum (**Supplementary Figure 1A**), jejunum (**Supplementary Figure 1B**), ileum (**Supplementary Figure 1C**), and colon (**Supplementary Figure 1D**) after 4 and 7 days of DSS treatment, but returned to normal levels after DSS was withdrawn.

#### Production of Inflammatory Mediators

**Figure 3:**
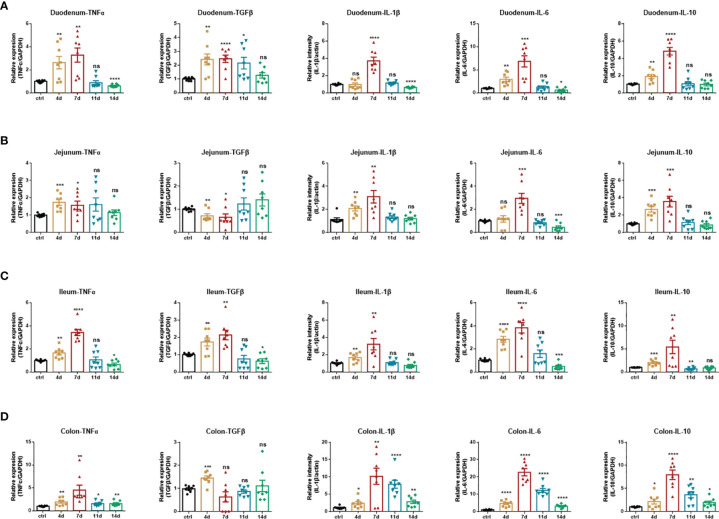
Inflammatory cytokines production of DSS-induced acute enteritis. **(A)** The mRNA expression levels of TNFα, TGFβ, IL-1β, IL-6, and IL-10 in duodenal tissues were detected by quantitative real‐time PCR. **(B)** The mRNA expression levels of TNFα, TGFβ, IL-1β, IL-6, and IL-10 in jejunal tissues were detected by quantitative real‐time PCR. **(C)** The mRNA expression levels of TNFα, TGFβ, IL-1β, IL-6, and IL-10 in ileal tissues were detected by quantitative real‐time PCR. **(D)** The mRNA expression levels of TNFα, TGFβ, IL-1β, IL-6, and IL-10 in colonic tissues were detected by quantitative real‐time PCR. The data were represented as mean ± SEM. n = 8 mice in each group. *P < 0.05, **P < 0.01, ***P < 0.001, ****P < 0.0001. ns, no significance.

Pro-inflammatory mediators play critical roles in the pathogenesis of IBD, namely, ulcerative colitis (UC) and Crohn’s diseases (CD). Enhanced intestinal permeability and subsequent immune cell infiltration are thought to increase the production of pro-inflammatory cytokines, both from immune cells and epithelial cells. Therefore, we employed real-time fluorescent quantitative PCR to study the production of inflammatory cytokines in each intestinal segment under DSS treatment. In the duodenum (**Figure 3A**), jejunum (**Figure 3B**), and ileum (**Figure 3C**), DSS significantly increased the production of inflammatory cytokines TNF-α, IL-1β, IL-6, and immunomodulatory cytokines TGF-β, IL-10. Furthermore, the production of these inflammatory cytokines progressively increased during DSS treatment and reached a maximum on the 7th day. After DSS withdrawal, they returned to normal levels. In the colon (**Figure 3D**), DSS also significantly increased the production of TNF-α, IL-1β, IL-6, and IL-10. The production of these cytokines progressively increased during DSS treatment and reached a maximum on the 7th day. However, after DSS withdrawal, these mediators were partially downregulated. Still, they remained at high levels until mice were sacrificed at day 14, which indicated that the duration of inflammation incited by DSS persists long, which implied the potential to switch to chronic inflammation.

Based on the findings above, we further detected and analyzed immune cell subsets in the intestinal tissues at the specified time (7 and 14 d). In DSS-induced acute enteritis, the results showed that macrophages in the intestinal tissues on the 7th and 14th days were 3.300 ± 0.03215% and 3.133 ± 0.06360%, respectively, which were both significantly lower than those in the control group (3.967 ± 0.07623%) (**Supplementary Figure 2A**). For CD4^+^ T cells, the 7th-day group (5.310 ± 0.1589%) was significantly higher than the control (4.013 ± 0.07219%), and the 14th-day group (4.427 ± 0.1444%) was not significantly different from the control (**Supplementary Figure 2B**). Moreover, for CD8^+^ T cells, the 7th-day group (14.82 ± 0.1354%) and the 14th-day group (14.60 ± 0.06807%) were significantly higher than the control group (10.21 ± 0.06692%) (**Supplementary Figure 2C**). The intrinsic lymphoid cells were significantly higher than the control group (11.13 ± 0.04372%) on the 7th day (16.61 ± 0.1562%), and significantly lower than the control group on the 14th day (9.397 ± 0.2317%) (**Supplementary Figure 2E**).

In summary, the data above indicate that the main affected area of intestinal inflammation induced by 3% DSS is colorectum, therefore we highly recommend the selection of this model when focusing on colorectal lesions.

### Anti-CD3 Antibody-Induced Hyperacute Enteritis Model

#### Weight Loss, Disease Index Score, and Bowel Length Shortening

**Figure 4:**
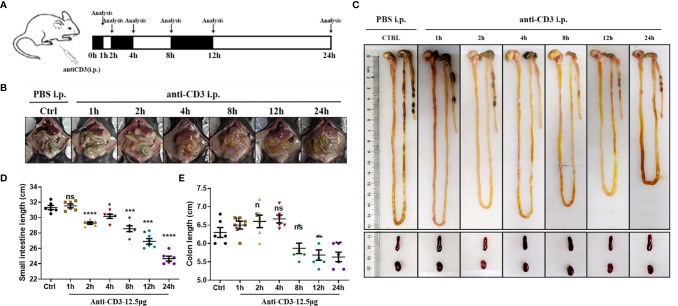
Observations of anti-CD3 antibody-induced acute enteritis. **(A)** Mice were intraperitoneally administered 100 μl of phosphate buffered saline (PBS) containing 12.5 μg of anti-CD3 antibody. The mice were sacrificed at 1, 2, 4, 8, 12, and 24 h after the anti-CD3 antibody treatment. **(B)** Representative schematics of the appearances of the small intestines in mice sacrificed at different hours. **(C)** Representative schematics of the whole intestine in mice sacrificed at different hours. **(D)** Small intestine lengths from stomach to cecum. **(E)** Colon lengths from cecum to anus. Data were represented as mean ± SEM. n = 6 mice in each group. *P < 0.05, **P < 0.01, ***P < 0.001, ****P < 0.0001. ns, no significance.

We induced experimental hyperacute enteritis in C57BL/6 mice by intraperitoneal injection of anti-CD3 antibody for the indicated time (**Figure 4A**). With the progress of anti-CD3 antibody treatment, mice showed a varying extent of diarrhea, intestinal edema, and shortened bowel length. After anti-CD3 antibody treatment, the mouse feces gradually became thinner, and there were no formed feces after 4 h. However, the symptoms gradually alleviated as time passed, and the feces were re-formed after 24 h (**Figures 4B, C**). Small intestine length and colon length were also measured to determine the severity of enteritis. Small intestine length shortening was significant and became more severe with time in anti-CD3 antibody-treated mice than in the small intestine from control mice injected with PBS intraperitoneally (**Figure 4D**). After 24 h, the length of the small intestine was reduced by about 21%. Colon length shortening was not signed until 12 h later in anti-CD3 antibody-treated mice (**Figure 4E**). Compared with the control mice, the colon length after 24 h from mice receiving anti-CD3 antibody treatment was only reduced by about 10%. Collectively, these data indicated that anti-CD3 antibody treatment could not only cause a significant reduction in the length of the small intestine, but only caused a slight impact on colon length.

#### Histological Characterization

**Figure 5:**
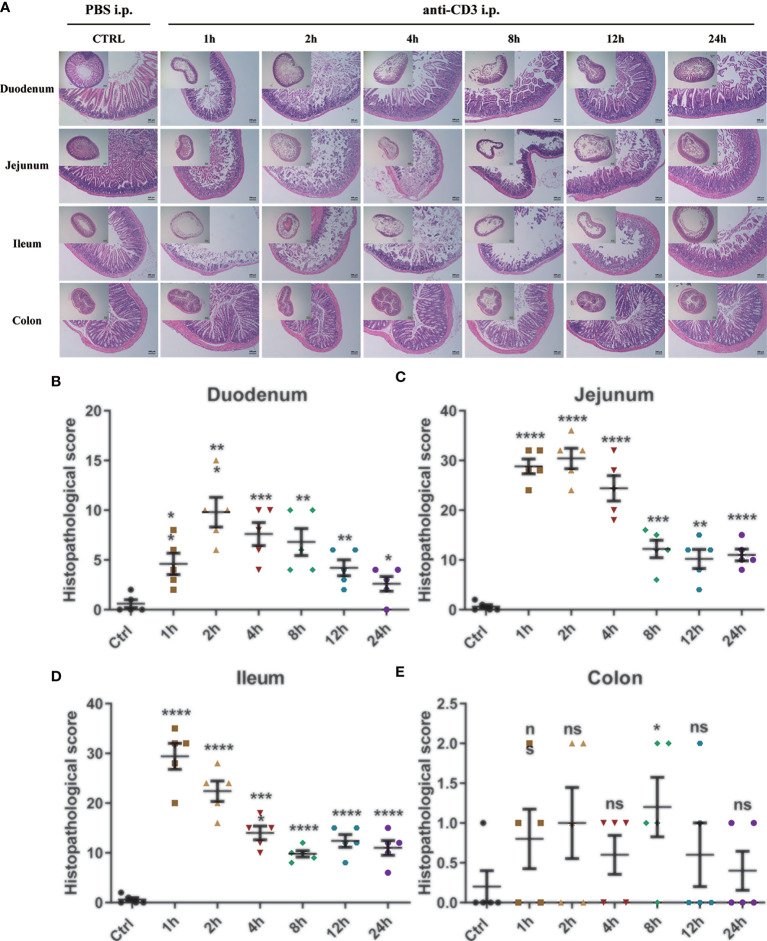
Histological observation of anti-CD3 antibody-induced acute enteritis. **(A)** Representative paraffin sections of duodenal, jejunal, ileal, and colonic tissues stained with H&E. Bars indicate 100 μm. **(B–E)** Histopathological scores of duodenal **(B)**, jejunal **(C)**, ileal **(D)**, and colonic **(E)** tissue sections (histological score = (inflammation + depth of lesions + crypt damage) × width of lesions.). Data were represented as mean ± SEM. n = 5 mice in each group. *P < 0.05, **P < 0.01, ***P < 0.001, ****P < 0.0001. ns, no significance.

Then, H&E staining and histopathological score were also performed to detect the tissue morphology and inflammatory infiltration in the duodenum, jejunum, ileum, and colon. The control mice’s duodenum, jejunum, ileum, and colon sections all showed intact epithelium structures, no ulcers or erosions, and no inflammatory cell infiltration in mucosa and submucosa. Duodenum, jejunum, and ileum tissues from the anti-CD3 antibody-treated mice were significantly damaged in the epithelial structure right after anti-CD3 antibody treatment, and gradually recovered with time. Furthermore, the damage happened rapidly, and the inflammatory lesions occurred significantly as early as 1 h after administration. However, there was no obvious damage in colon tissues when mice were treated with the anti-CD3 antibody (**Figure 5A**). In duodenum and jejunum tissues from the anti-CD3 antibody-treated mice, the histopathological score increased to the highest in the 2 h group, then gradually decreased, but it was still higher than the control group (**Figures 5B, C**). In ileum tissues from the anti-CD3 antibody-treated mice, the histopathological score reached the maximum at 1 h, then gradually decreased as time passed (**Figure 5D**). In colon tissue from the anti-CD3 antibody-treated mice, the histopathological score was not significantly different among groups (**Figure 5E**). Moreover, we also detected the expression of lipocalin-2 in each intestinal segment. The results showed that lipocalin-2 in the intestinal tissue was significantly increased after the anti-CD3 treatment. Among them, lipocalin-2 in the duodenum (**Supplementary Figure 3A**) and jejunum (**Supplementary Figure 3B**) reached the highest value at 2 h, while lipocalin-2 in the ileum (**Supplementary Figure 3C**) and colon (**Supplementary Figure 3D**) had the highest expression at 4 h.

#### Production of Inflammatory Mediators

**Figure 6:**
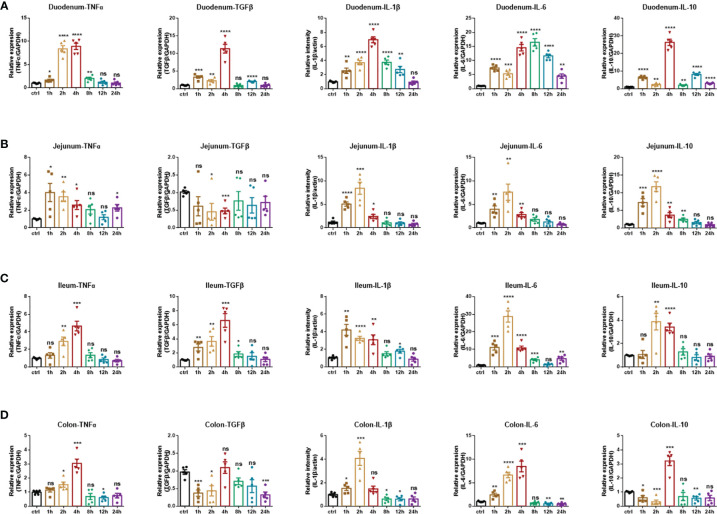
Inflammatory cytokines production of anti-CD3 antibody-induced acute enteritis. **(A)** The mRNA expression levels of TNFα, TGFβ, IL-1β, IL-6, and IL-10 in duodenal tissues were detected by quantitative real‐time PCR. **(B)** The mRNA expression levels of TNFα, TGFβ, IL-1β, IL-6, and IL-10 in jejunal tissues were detected by quantitative real‐time PCR. **(C)** The mRNA expression levels of TNFα, TGFβ, IL-1β, IL-6, and IL-10 in ileal tissues were detected by quantitative real‐time PCR. **(D)** The mRNA expression levels of TNFα, TGFβ, IL-1β, IL-6, and IL-10 in colonic tissues were detected by quantitative real‐time PCR. The data were represented as mean ± SEM. n = 5 mice in each group. *P < 0.05, **P < 0.01, ***P < 0.001, ****P < 0.0001. ns, no significance.

Consistently, to detect the production of inflammatory cytokines, namely, TNF-α, IL-1β, IL-6, and immunomodulatory cytokines TGF-β, IL-10 in each intestinal segment under the treatment of the anti-CD3 antibody, real-time fluorescent quantitative PCR analysis was also used. In the anti-CD3 antibody-treated duodenum tissue, the expression of TNF-α, TGF-β, IL-1β, and IL-10 showed an overall trend of increase initially, followed by a decrease, and reached the highest value at 4 h. Also, the expression of IL-6 showed a similar trend but reached the highest value at 8 h (**Figure 6A**). In the anti-CD3 antibody-treated jejunum tissue, the expression of IL-1β, IL-6, and IL-10 also showed an overall trend of increase first and then decreasing, and reached a maximum at 2 h. The expression of TNF-α showed a similar trend but reached the highest value rapidly at 1 h. The expression of TGF-β was significantly downregulated in the 2 h- and 4 h-groups, and there was no significant difference in other groups compared with the control group (**Figure 6B**). In the ileum, the anti-CD3 antibody treatment significantly increased the production of inflammatory cytokines TNF-α, TGF-β, IL-1β, IL-6, and IL-10. The production of these inflammatory cytokines all showed the same trend as in duodenum tissues. Among them, IL-1β reached a maximum at 1 h, IL-6 and IL-10 reached a maximum at 2 h, and TNF-α and TGF-β reached a maximum at 4 h. In addition, the expression of TGF-β was significantly downregulated in the 2 h-, 4 h- and 24 h-groups, and there was no significant difference in other groups compared with the control group (**Figure 6C**). In the anti-CD3 antibody-treated colon tissue, the expression of TNF-α, TGF-β, IL-6, and IL-10 showed an overall trend of increase first and then decreasing, and reached the highest value at 4 h. The expression of IL-1β showed a similar trend but reached a maximum at 2 h (**Figure 6D**). All these mediators in each intestinal tissue returned to normal or remained low levels after 24 h of the anti-CD3 antibody treatment, indicating that the inflammation caused by the anti-CD3 antibody treatment had a short duration and was easy to recover.

Otherwise, to better understand the process of enteritis, we also detected the immune cell subpopulations in the intestinal tissue at the specified time (2 and 24 h). In hyperacute enteritis induced by the anti-CD3 antibody, CD4^+^ T cells in the intestinal tissue at 2 h (6.753 ± 0.09838%) and 24 h (6.080 ± 0.05292%) were significantly higher than those in the control (5.337 ± 0.05783%) (**Supplementary Figure 4B**). For CD8^+^ T cells, CD8^+^ T cells in the intestinal tissue at 2 h (6.557 ± 0.1277%) and 24 h (4.643 ± 0.1244%) were significantly lower than those in the control (8.523 ± 0.08090%) (**Supplementary Figure 4C**). For neutrophils, 2 h-treated group (2.777 ± 0.1040%) was significantly higher than the control (1.233 ± 0.04410%), and 24 h-treated group (1.580 ± 0.1582%) was not significantly different from the control (**Supplementary Figure 4D**). The intrinsic lymphoid cells were significantly higher than the control group (4.603 ± 0.2784%) at 2 h (14.58 ± 0.1997%), and significantly lower than the control group at 24 h (3.597 ± 0.1426%) (**Supplementary Figure 4E**).

In a nutshell, this part of our data shows that in the anti-CD3 antibody-induced enteritis model, the damage is concentrated in the small intestine, especially the jejunum segment. Therefore, it is recommended that this model should be given priority in the related research on the small intestine.

#### Dextran Sodium Sulfate (DSS)-Induced Chronic Enteritis Model

**Figure 7:**
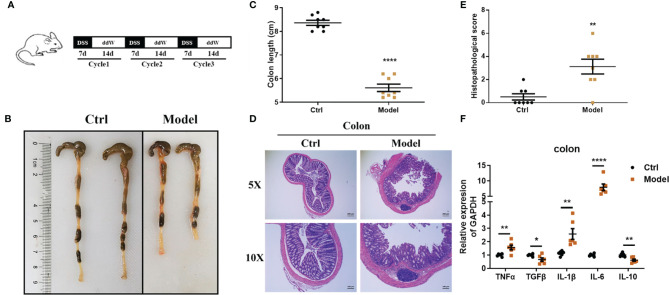
Observations of DSS-induced chronic enteritis. **(A)** Mice were fed with 3 cycles of DSS treatment (including 7 days with 1.5% DSS in sterile water followed by a 14-day recovery phase with sterile water). **(B)** Representative schematics of the colon in mice sacrificed at the end of the experiment. **(C)** Colon lengths from cecum to anus. **(D)** Representative paraffin sections of colonic tissues stained with H&E. Bars indicate 100 μm. **(E)** Histopathological scores of colonic tissue sections. **(F)** The mRNA expression levels of TNFα, TGFβ, IL-1β, IL-6, and IL-10 in colonic tissues were detected by quantitative real‐time PCR (n = 5). Data were represented as mean ± SEM. n = 8 mice in each group. *P < 0.05, **P < 0.01, ****P < 0.0001.

IBD is characterized by chronic inflammatory responses and multiple exacerbations during disease progression, and previous results showed that in DSS-induced acute enteritis, inflammatory cytokines maintained high levels and lasted for a long time after DSS withdrawal. Therefore, we constructed a mouse model of chronic colitis in C57BL/6 mice through 3 cycles of DSS treatment as described in the *Materials and Methods* (**Figure 7A**). Under these conditions, chronic colitis developed, accompanied by reduced colon length, similar to that observed in DSS-induced acute colitis (**Figures 7, C**). In accordance with these signs of colitis, mice further exhibited pathological characteristics at the endpoint of the experiment, which showed necrosis, ulcerations, infiltration of inflammatory cells into the colonic mucosal and submucosal, and obvious thickening of the mucosa and muscle layer (**Figure 7D**).

Consistent with this, the histopathological score of the model group was significantly higher than that of the control group (**Figure 7E**). Likewise, the level of lipocalin-2 mRNA increased significantly in the colon (**Supplementary Figure 5A**). We further employed real-time fluorescent quantitative PCR to study the production of inflammatory cytokines TNF-α, IL-1β, IL-6, and immunomodulatory cytokines TGF-β, IL-10 in colon tissue under this treatment. Compared with the control group, TNF-α, IL-1β, and IL-6 were significantly upregulated in the model group, while TGF-β and IL-10 were significantly downregulated (**Figure 7F**). Next, we also detected the immune cell subsets in the intestinal tissue. In DSS-induced chronic enteritis, CD4^+^T cells in the intestinal tissue of the model group (4.407 ± 0.09528%) were significantly lower than those in the control group (5.277 ± 0.07265%) (**Supplementary Figure 6B**). CD8^+^ T cells in the intestinal tissue of the model group (2.413 ± 0.2360%) were also significantly lower than the control (3.537 ± 0.06741%) (**Supplementary Figure 6D**). In addition, macrophages (**Supplementary Figure 6A**), neutrophils (**Supplementary Figure 6C**), and intrinsic lymphoid cells (**Supplementary Figure 6E**) in the intestinal tissues of the model group were not significantly different from those of the control group.

Therefore, in the DSS-induced chronic enteritis model, we also recommend a major focus on colorectal lesions.

#### High-Fat Feed (HFD)-Induced Chronic Enteritis Model

**Figure 8:**
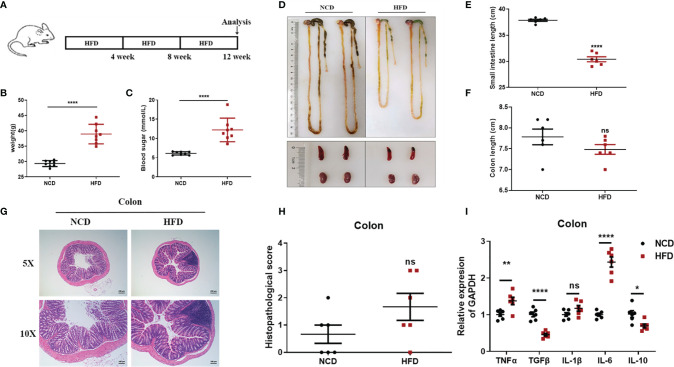
Observations of HFD-induced chronic enteritis. **(A)** Mice were fed with HFD for 12 weeks to induce chronic enteritis in C57BL/6 mice. **(B)** Bodyweight detection of both groups at the end of the experiment (n = 8). **(C)** Blood glucose concentration detection of both groups at the end of the experiment (n = 8). **(D)** Representative schematic of the whole intestine in mice sacrificed at the end of the experiment. **(E)** Small intestine lengths from stomach to cecum. **(F)** Colon lengths from cecum to anus. **(G)** Representative paraffin sections of colonic tissues stained with H&E. Bars indicate 100 μm. **(H)** Histopathological scores of colonic tissue sections. **(I)** The mRNA expression levels of TNFα, TGFβ, IL-1β, IL-6, and IL-10 in colonic tissues were detected by quantitative real‐time PCR. Data were represented as mean ± SEM. n = 6 mice in each group. *P < 0.05, **P < 0.01, ***P < 0.001, ****P < 0.0001. ns, no significance.

Mounting evidence links high-fat feeding (HFD) to low-grade intestinal inflammation (26, 27). Therefore, we also induced chronic enteritis in C57BL/6 mice through high-fat feeding for 12 weeks to compare with chronic enteritis induced by DSS, and details were shown in **Figure 8A**. As shown in **Figures 8B, C**, after 12 weeks of high-fat diet feeding, the body weight and blood glucose concentration levels of the HFD mice increased significantly, which were about twice as high as those of the NCD mice. However, in this model, we did not observe a significant reduction in colon length, but a significant shortening of the small intestine (**Figures 8D–F**). Similarly, we did not observe obvious necrosis, ulceration, or inflammatory cell infiltration in the colon at the end of the experiment (**Figure 8G**). Also, there was no significant difference in histopathological scores compared with the control (**Figure 8H**). Due to the obvious shortening of the small intestine, H&E staining was used to further indicate the histomorphology and inflammatory infiltration in the duodenum, jejunum, and ileum, and the histopathological score was also performed. We observed that the duodenum of HFD-fed mice had no obvious damage (**Supplementary Figure 7A**), while the jejunum and ileum tissues were more severe and extensive, with prominent mucosal erosions and crypt abscesses (**Supplementary Figures 7B, C**). In the jejunum and ileum, compared with the control group, the pathology score of the HFD-fed group was also significantly higher, while there was no significant difference in the duodenum (**Supplementary Figures 7D–F**). Similarly, we detected the lipocalin-2 mRNA level in each intestinal segment by qPCR. As shown in **Supplementary Figure 8**, lipocalin-2 expression levels increased significantly in the duodenum (**Supplementary Figure 8A**), jejunum (**Supplementary Figure 8B**), ileum (**Supplementary Figure 8C**) and colon (**Supplementary Figure 8D**) after HFD treatment.

We further employed real-time fluorescent quantitative PCR to detect the production of inflammatory cytokines TNF-α, IL-1β, IL-6, and immunomodulatory cytokines TGF-β, IL-10 in each intestinal tissue after an NCD or an HFD feeding. In the colon, compared to the control group, TNF-α and IL-6 were significantly upregulated in the HFD-fed group, while TGF-β and IL-10 were significantly downregulated (**Figure 8I**). In the duodenum, compared to the control group, IL-1β was significantly upregulated in the HFD-fed group, while TGF-β and IL-10 were significantly downregulated (**Supplementary Figure 7G**). In the jejunum, compared with the control group, TGF-β, IL-1β, and IL-6 were significantly upregulated in the HFD-fed group, while IL-10 was significantly downregulated (**Supplementary Figure 7H** **)**. In the ileum, compared with the control group, only IL-10 was significantly downregulated in the HFD-fed group (**Supplementary Figure 7I**). However, immune cell subsets in the intestinal tissues of HFD-induced chronic enteritis were also detected. As shown in **Supplementary Figure 9B**, CD4^+^T cells in the intestinal tissue of the model group (4.303 ± 0.05364%) were significantly lower than those in the control group (6.430 ± 0.1229%). While, macrophages, CD8^+^ T cells, neutrophils and intrinsic lymphoid cells in the intestinal tissue of the model group were not significantly different from those of the control group (**Supplementary Figures 9A, C–E**).

To sum up, our data indicate that in the intestinal inflammation complicated by high-fat feeding, attention should be paid to the injury of the small intestine, whether it is for therapeutic research or mechanism exploration.

#### Intestinal Microbial Detection of Four Enteritis Models

In recent years, several studies have found that intestinal flora plays an important role in the occurrence and development of enteritis (2, 38, 39). Therefore, we also tested the intestinal flora in each model. As shown in **Supplementary Figure 10A**, in DSS-induced acute enteritis, the fecal microbial structure of the mice was significantly changed between the 7 and 14 d and the pre-modeling (0 d) mice, showing different bacterial phylum abundances. We observed that DSS increased the ratio of Firmicutes and Proteobacteria, and the increase in the ratio of Firmicutes was a marker of gut dysbiosis (40). In hyperacute enteritis induced by an anti-CD3 antibody, the proportion of Firmicutes decreased at 2 h, but increased at 24 h (**Supplementary Figure 10B**). In DSS-induced chronic enteritis, there were also significant differences in the microbial structure of mice feces before and after the model was established. DSS increased the proportion of Firmicutes, Desulfobacterota, and Proteobacteria (**Supplementary Figure 10C**). In HFD-induced chronic enteritis, the microbial structure of the feces of mice also changed significantly before and after the establishment of the model. HFD increased the proportion of Firmicutes, Desulfobacterota, Proteobacteria, and Campilobecterota (**Supplementary Figure 10D**).

## Discussion

Our research included a detailed analysis of clinical, histological parameters, and cytokine profiles in the duodenum, jejunum, ileum, and colon in a spatiotemporal manner during acute enteritis and recovery stage. Clinical and histological changes were determined based on phenotypic and pathologic changes (25, 41, 42), such as diarrhea, rectal bleeding, bodyweight loss, and colon shortening, which are the common phenomena seen in DSS-induced experimental enteritis. Diarrhea is caused by the increased permeability of intestinal cells or intraluminal hypertonicity led by DSS (43, 44). Weight loss and colon shortening, as indicators for the severity of intestinal inflammation, correlate with the pathological and histological changes and are consistent markers for colitis (5).

**Figure 9:**
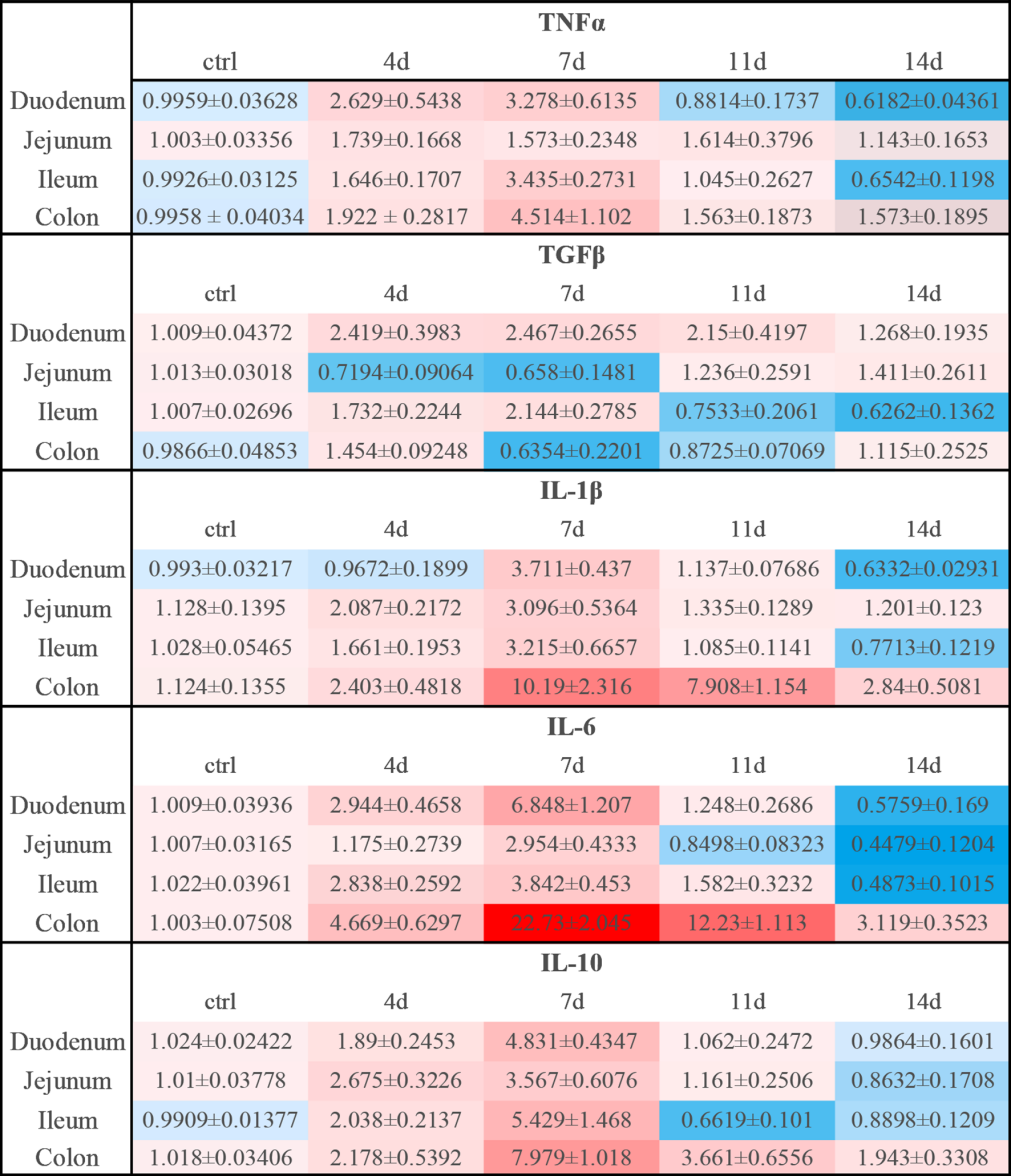
Relative expression levels of inflammatory cytokines in DSS-induced acute enteritis. In DSS-induced acute enteritis, we used qPCR to compare the expression levels of inflammatory cytokines in different intestinal segments on different modeling days, and the colon was confirmed as our control group in the statistical analysis and comparison. Among them, red indicates that the expression level is higher than the expression level in the colon, blue indicates that the expression level is lower than the expression level in the colon. The shade of the color red/blue represents a relative degree of upward or downward adjustment. Results expressed as Mean ± SEM. n = 8 mice in each group.

**Figure 10:**
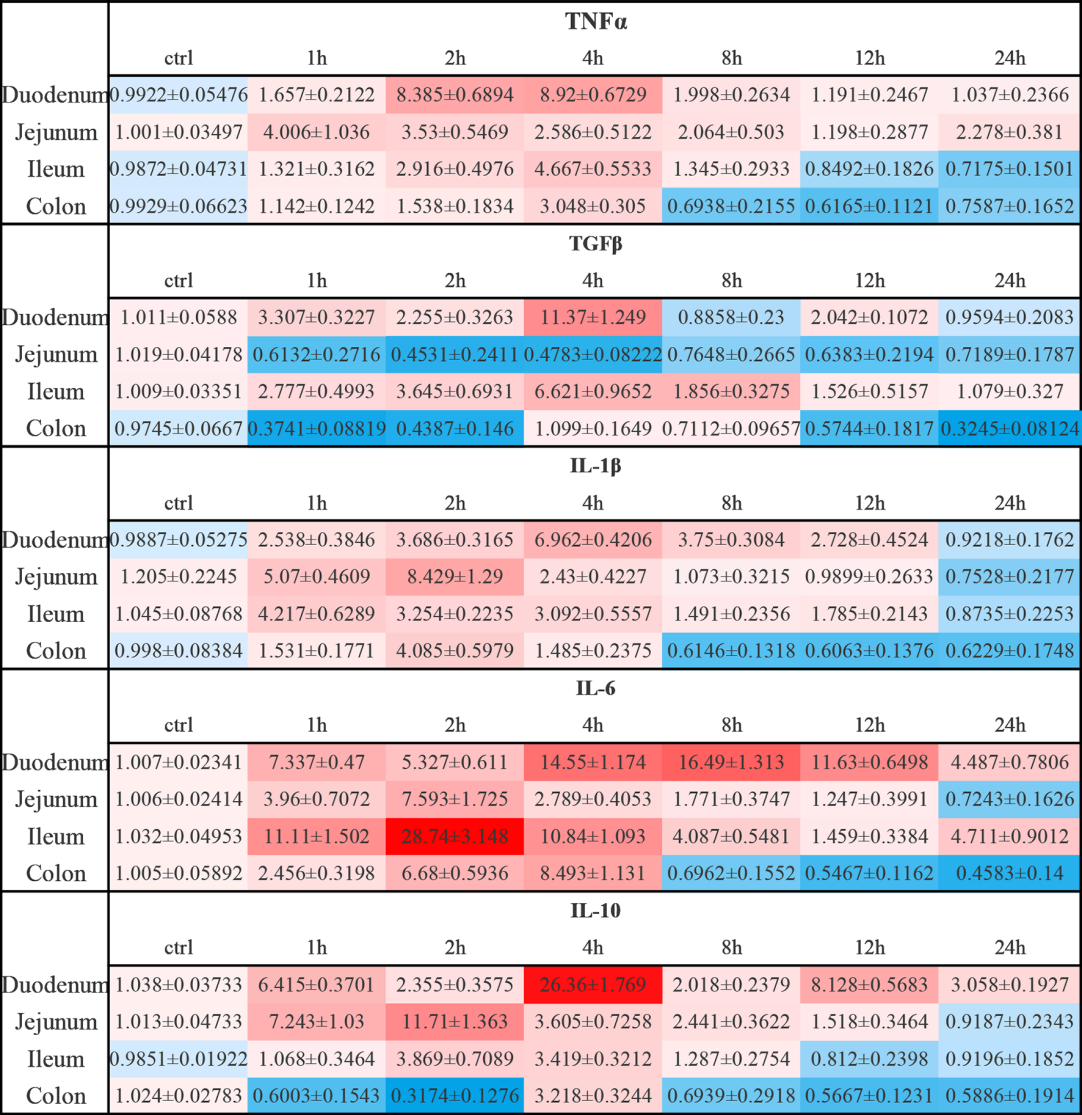
Relative expression levels of inflammatory cytokines in anti-CD3 antibody-induced acute enteritis. In anti-CD3 antibody-induced acute enteritis, we used qPCR to compare the expression levels of inflammatory cytokines in each segment of the intestine at different detection times after anti-CD3 antibody treatment, and the colon was confirmed as our control group in the statistical analysis and comparison. Among them, red indicates that the expression level is higher than the expression level in the colon, blue indicates that the expression level is lower than the expression level in the colon. The shade of the color red/blue represents a relative degree of upward or downward adjustment. Results expressed as Mean ± SEM. n = 6 mice in each group.

In DSS-induced acute enteritis, our results suggested that weight loss, disease index score, and bowel length shortening all became severe during the DSS treatment, and then attenuated after DSS withdrawal. However, the maximum shortening of the colon (28%) after 7 days of DSS treatment was significantly higher than that of the small intestine (20%). Based on this, we observed the injury of each intestinal segment by H&E staining, and the overall situation also showed a trend of aggravation of the injury during the DSS induction period as time prolonged in the recovery period. Collectively, the serious damage caused by DSS mainly occurred in the colon, accompanied slight damage in the ileum, and no obvious damage in the duodenum and jejunum. In the anti-CD3 antibody-induced acute enteritis, our results also showed that diarrhea was obvious at the initial stage of anti-CD3 antibody treatment, and then gradually recovered with time. However, the shortening of intestinal length gradually increased with time in the anti-CD3 antibody-treated mice. Interestingly, unlike DSS-induced acute enteritis, the maximum shortened length of the small intestine (21%) in acute enteritis induced by the anti-CD3 antibody was significantly higher than that of the colon (10%). The injury of each intestine segment observed by H&E also showed that the injury of each intestine segment (namely, the duodenum, jejunum, and ileum) of the small intestine was significantly higher compared with that of the control mice, while no significant difference was observed in the colon between anti-CD3 antibody-treated mice and control mice. In addition, pro-inflammatory cytokines are local inflammatory mediators, produced by macrophages, lymphocytes and also epithelial and mesenchymal cells, participating in the development and pathogenesis of inflammation and immunity (45). There was enhanced inflammatory mediators production in each intestinal segment of the DSS-treated mice. We found that the duodenum, jejunum, ileum, and colon express different levels of pro-inflammatory mediators. Generally, after DSS treatment, the distal colon produced much higher levels of pro-inflammatory mediators, especially IL-1β and IL-6 (**Figure 9**, **Supplementary Table S2**). In the duodenum, jejunum and ileum, almost all tested cytokine levels during the recovery phase returned to baseline or even lower, while the levels of these cytokines in the colon were still relatively high (**Figures 3**, **9**, **Supplementary Table S2**). This was in keeping with the severe histological damage in the colon compared with the small intestine. These results indicate that the colon is more affected by the DSS, which is consistent with previous research data. Our further detection of immune cells showed that DSS treatment mainly increased the proportion of CD4^+^T cells, CD8^+^T cells, and ILC cells in the intestinal tissue. The production of inflammatory mediators in each intestinal segment of mice treated with anti-CD3 antibody was also enhanced, and the expression levels were different in each intestinal segment. After anti-CD3 antibody treatment, we found that higher levels of pro-inflammatory mediators such as TNFα, IL-1β, and IL-6 were produced in the small intestine instead of the colon (**Figure 10**, **Supplementary Table S3**), indicating that the small intestine is more susceptible to anti-CD3 antibody treatment. Our further flow cytometry results indicated that the anti-CD3 antibody treatment mainly increased the proportion of CD4^+^T cells, CD8^+^T cells, neutrophils, and ILC cells in the intestinal tissue. In addition, both DSS and anti-CD3 treatments increased the proportion of Firmicutes in the intestinal flora. Altogether, these results indicate that in 3% DSS-induced acute enteritis, the colorectal injury is significantly higher than that of the small intestine, while in anti-CD3 antibody-induced acute enteritis, the small intestine injury is significantly higher than that of the colon.

In the DSS-induced chronic enteritis, we also observed a significant decrease in the colon length compared with that of control mice (8.4%), but it was significantly lower than the decrease in colon length (28%) in the DSS-induced acute enteritis. H&E observation also showed that the colon injury of mice in the chronic enteritis group induced by DSS was more serious than that of the control mice. At the same time, significant upregulation of TNF-α, IL-1β, and IL-6, and significant downregulation of TGF-β and IL-10 were detected in the experimental group. Our further examination of immune cells showed that the DSS treatment mainly increased the proportion of CD4^+^T cells and CD8^+^T cells in the intestinal tissue. Unlike this, in the HFD-induced chronic enteritis, the colon length and histopathological score of the experimental group were not significantly different from those of the control mice. However, we observed that the long-term administration of HFD caused a significant reduction in the length of the small intestine. Further H&E observations found that the small intestine, especially the jejunum and ileum, was significantly damaged. The HFD group had been shown to increase the expression of pro-inflammatory cytokines in the colon (26–28). Consistent with this, in the detection of cytokines, we found that TNF-α and IL-6 were upregulated in the colon of the model group. At the same time, we found that the HFD administration also caused significant upregulation of TGF-β, IL-1β, and IL-6 in the jejunum, but IL-6 in the jejunum was upregulated more significantly than in the colon. Our further flow cytometry results indicated that HFD treatment mainly increased the proportion of CD4^+^ T cells in the intestinal tissue. In addition, both the DSS and HFD treatments increased the proportion of Firmicutes, Desulfobacterota and Proteobacteria in the intestinal flora. The above results indicate that in the 1.5% DSS-induced chronic enteritis, the damage is mainly concentrated in the colorectal, while the damage caused by long-term HFD-induced chronic enteritis is more focused on the small intestine.

In conclusion, we conducted a rigorous analysis of several enteritis modeling methods and correlated the cytokine profile with clinical and histological parameters. Additionally, our data provide novel insight into the differential expression of cytokine, especially IL-1β and IL-6, in the duodenum, jejunum, ileum, and colon, providing a reference for model selection to analyze the role of these cytokines in inducing and recovering from inflammation. Besides, our results indicate that different modeling methods have a preference for the site of intestinal injuries. We suggest that when studying the pathogenesis of enteritis or evaluating the efficacy of drugs, appropriate models should be selected for research purposes. Moreover, the occurrence of the disease is a complex process with multiple factors. Combining different models to simulate clinical features better has also attracted increasing attention. For example, a high-fat and/or high-sugar diet has been shown to cause low-grade intestinal inflammation in mice (26–28, 46). HFD has been shown to exacerbate DSS colitis by upregulating pro-inflammatory cytokines (47, 48) and exacerbating mucosal tissue damage in mouse models of spontaneous colitis (49) and ileitis (50, 51). Therefore, our results also provide a certain reference for the combined research of enteritis models. We look forward to more development or combination research of enteritis models to provide more insights into the histopathological and morphological changes and factors associated with the pathogenesis of IBD, which will be a benefit for precise treatment.

## Data Availability Statement

The original contributions presented in the study are included in the article/**Supplementary Material**. Further inquiries can be directed to the corresponding authors.

## Ethics Statement

The animal study was reviewed and approved by the Nanjing University Animal Care and Use Committee.

## Author Contributions

Z-CH and HZ designed the outline of the paper. HZ revised this manuscript. HX and FC contributed equally to this work. HX and FC performed most of the experiments in this study. HX wrote the manuscript and prepared the figures. PL, XW, YY, XC, ZB, and HS helped with the molecular biology related experiments. PL and HS helped with the experiments using animals. All authors contributed to the article and approved the submitted version.

## Funding

This study was supported by grants from the Chinese National Natural Sciences Foundation (81773099 and 82130106), the National Key R&D Program of China (2017YFA0506002), and the Jiangsu Province Natural Sciences Foundation (BK20192005).

## Conflict of Interest

Author Z-CH was employed by Jiangsu TargetPharma Laboratories Inc.

The remaining authors declare that the research was conducted in the absence of any commercial or financial relationships that could be construed as a potential conflict of interest.

## Publisher’s Note

All claims expressed in this article are solely those of the authors and do not necessarily represent those of their affiliated organizations, or those of the publisher, the editors and the reviewers. Any product that may be evaluated in this article, or claim that may be made by its manufacturer, is not guaranteed or endorsed by the publisher.
