# Supplementary material for: Characterization and Analysis of the Temporal and Spatial Dynamic of Several Enteritis Modeling Methodologies
Source: Front Immunol. 2021 Dec 22;12:727664. doi: 10.3389/fimmu.2021.727664 (PMC8728876; doi:10.3389/fimmu.2021.727664)
Supplement: Supplementary file 1 [file DataSheet_1.docx]

**Table S1: The primers of selected genes for real time PCR.**

| **Gene** | **Forward primer (5’-3’)** | **Reverse primer (5’-3’)** |
| --- | --- | --- |
| **TNFα** | CGTCAGCCGATTTGCTATCT | CGGACTCCGCAAAGTCTAAG |
| **TGFβ** | GGAGGTACCGCCCGGCCCGC | GACAGCAATGGGGGTTCGGG |
| **IL-1β** | GAGAGCCGGGTGACAGTATC | TGACAAACTTCTGCCTGACG |
| **IL-6** | AGTTGCCTTCTTGGGACTGA | CAGAATTGCCATTGCACAAC |
| **IL-10** | CCAAGCCTTATCGGAAATGA | TTTTCACAGGGGAGAAATCG |
| **GAPDH** | CTTCACCACCATGGAGGAGGC | GGCATGGACTGTGGTCATGAG |
| **Lipocalin-2** | TGGCCCTGAGTGTCATGTG | CTCTTGTAGCTCATAGATGGTGC |
| **Actin** | GAGACCTTCAACACCCCAGC | ATGTCACGCACGATTTCCC |

**Table S2: Relative expression levels of inflammatory cytokines in DSS-induced acute enteritis**

|  | **TNFα** | | | | |
| --- | --- | --- | --- | --- | --- |
|  | **ctrl** | **4d** | **7d** | **11d** | **14d** |
| **Duodenum** | 0.9959±0.03628 | 2.629±0.5438 | 3.278±0.6135 | 0.8814±0.1737^a^ | 0.6182±0.04361^c^ |
| **Jejunum** | 1.003±0.03356 | 1.739±0.1668 | 1.573±0.2348^a^ | 1.614±0.3796 | 1.143±0.1653 |
| **Ileum** | 0.9926±0.03125 | 1.646±0.1707 | 3.435±0.2731 | 1.045±0.2627 | 0.6542±0.1198^b^ |
| **Colon** | 0.9958 ± 0.04034 | 1.922 ± 0.2817 | 4.514±1.102 | 1.563±0.1873 | 1.573±0.1895 |
|  | **TGFβ** | | | | |
|  | **ctrl** | **4d** | **7d** | **11d** | **14d** |
| **Duodenum** | 1.009±0.04372 | 2.419±0.3983^a^ | 2.467±0.2655^c^ | 2.15±0.4197^b^ | 1.268±0.1935 |
| **Jejunum** | 1.013±0.03018 | 0.7194±0.09064^d^ | 0.658±0.1481 | 1.236±0.2591 | 1.411±0.2611 |
| **Ileum** | 1.007±0.02696 | 1.732±0.2244 | 2.144±0.2785^c^ | 0.7533±0.2061 | 0.6262±0.1362 |
| **Colon** | 0.9866±0.04853 | 1.454±0.09248 | 0.6354±0.2201 | 0.8725±0.07069 | 1.115±0.2525 |
|  | **IL-1β** | | | | |
|  | **ctrl** | **4d** | **7d** | **11d** | **14d** |
| **Duodenum** | 0.993±0.03217 | 0.9672±0.1899^a^ | 3.711±0.437^a^ | 1.137±0.07686^d^ | 0.6332±0.02931^c^ |
| **Jejunum** | 1.128±0.1395 | 2.087±0.2172 | 3.096±0.5364^b^ | 1.335±0.1289^d^ | 1.201±0.123^b^ |
| **Ileum** | 1.028±0.05465 | 1.661±0.1953 | 3.215±0.6657^a^ | 1.085±0.1141^d^ | 0.7713±0.1219^b^ |
| **Colon** | 1.124±0.1355 | 2.403±0.4818 | 10.19±2.316 | 7.908±1.154 | 2.84±0.5081 |
|  | **IL-6** | | | | |
|  | **ctrl** | **4d** | **7d** | **11d** | **14d** |
| **Duodenum** | 1.009±0.03936 | 2.944±0.4658^a^ | 6.848±1.207^d^ | 1.248±0.2686^d^ | 0.5759±0.169^d^ |
| **Jejunum** | 1.007±0.03165 | 1.175±0.2739^c^ | 2.954±0.4333^d^ | 0.8498±0.08323^d^ | 0.4479±0.1204^d^ |
| **Ileum** | 1.022±0.03961 | 2.838±0.2592^a^ | 3.842±0.453^d^ | 1.582±0.3232^d^ | 0.4873±0.1015^d^ |
| **Colon** | 1.003±0.07508 | 4.669±0.6297 | 22.73±2.045 | 12.23±1.113 | 3.119±0.3523 |
|  | **IL-10** | | | | |
|  | **ctrl** | **4d** | **7d** | **11d** | **14d** |
| **Duodenum** | 1.024±0.02422 | 1.89±0.2453 | 4.831±0.4347^a^ | 1.062±0.2472^b^ | 0.9864±0.1601^a^ |
| **Jejunum** | 1.01±0.03778 | 2.675±0.3226 | 3.567±0.6076^b^ | 1.161±0.2506^b^ | 0.8632±0.1708^a^ |
| **Ileum** | 0.9909±0.01377 | 2.038±0.2137 | 5.429±1.468 | 0.6619±0.101^c^ | 0.8898±0.1209^b^ |
| **Colon** | 1.018±0.03406 | 2.178±0.5392 | 7.979±1.018 | 3.661±0.6556 | 1.943±0.3308 |

Results expressed as Mean ± SEM. n = 8 mice in each group. The statistical analysis and comparison used the colon in each group as a control. *^a^P < 0.05*, *^b^P < 0.01*, *^c^P < 0.001*, *^d^P < 0.0001* (Student's t-test).

**Table S3: Relative expression levels of inflammatory cytokines in anti-CD3 Antibody–induced acute enteritis**

|  | **TNFα** | | | | | | |
| --- | --- | --- | --- | --- | --- | --- | --- |
|  | **ctrl** | **1h** | **2h** | **4h** | **8h** | **12h** | **24h** |
| **Duodenum** | 0.9922±0.05476 | 1.657±0.2122 | 8.385±0.6894^d^ | 8.92±0.6729^d^ | 1.998±0.2634^b^ | 1.191±0.2467 | 1.037±0.2366 |
| **Jejunum** | 1.001±0.03497 | 4.006±1.036^a^ | 3.53±0.5469^b^ | 2.586±0.5122 | 2.064±0.503^a^ | 1.198±0.2877 | 2.278±0.381^b^ |
| **Ileum** | 0.9872±0.04731 | 1.321±0.3162 | 2.916±0.4976^a^ | 4.667±0.5533^a^ | 1.345±0.2933 | 0.8492±0.1826 | 0.7175±0.1501 |
| **Colon** | 0.9929±0.06623 | 1.142±0.1242 | 1.538±0.1834 | 3.048±0.305 | 0.6938±0.2155 | 0.6165±0.1121 | 0.7587±0.1652 |
|  | **TGFβ** | | | | | | |
|  | **ctrl** | **1h** | **2h** | **4h** | **8h** | **12h** | **24h** |
| **Duodenum** | 1.011±0.0588 | 3.307±0.3227^d^ | 2.255±0.3263^c^ | 11.37±1.249^d^ | 0.8858±0.23 | 2.042±0.1072^c^ | 0.9594±0.2083^a^ |
| **Jejunum** | 1.019±0.04178 | 0.6132±0.2716 | 0.4531±0.2411 | 0.4783±0.08222^b^ | 0.7648±0.2665 | 0.6383±0.2194 | 0.7189±0.1787 |
| **Ileum** | 1.009±0.03351 | 2.777±0.4993^b^ | 3.645±0.6931^b^ | 6.621±0.9652^c^ | 1.856±0.3275^a^ | 1.526±0.5157 | 1.079±0.327 |
| **Colon** | 0.9745±0.0667 | 0.3741±0.08819 | 0.4387±0.146 | 1.099±0.1649 | 0.7112±0.09657 | 0.5744±0.1817 | 0.3245±0.08124 |
|  | **IL-1β** | | | | | | |
|  | **ctrl** | **1h** | **2h** | **4h** | **8h** | **12h** | **24h** |
| **Duodenum** | 0.9887±0.05275 | 2.538±0.3846^a^ | 3.686±0.3165 | 6.962±0.4206^d^ | 3.75±0.3084^d^ | 2.728±0.4524^b^ | 0.9218±0.1762 |
| **Jejunum** | 1.205±0.2245 | 5.07±0.4609^d^ | 8.429±1.29^a^ | 2.43±0.4227 | 1.073±0.3215 | 0.9899±0.2633 | 0.7528±0.2177 |
| **Ileum** | 1.045±0.08768 | 4.217±0.6289^b^ | 3.254±0.2235 | 3.092±0.5557^a^ | 1.491±0.2356^a^ | 1.785±0.2143^b^ | 0.8735±0.2253 |
| **Colon** | 0.998±0.08384 | 1.531±0.1771 | 4.085±0.5979 | 1.485±0.2375 | 0.6146±0.1318 | 0.6063±0.1376 | 0.6229±0.1748 |
|  | **IL-6** | | | | | | |
|  | **ctrl** | **1h** | **2h** | **4h** | **8h** | **12h** | **24h** |
| **Duodenum** | 1.007±0.02341 | 7.337±0.47^d^ | 5.327±0.611 | 14.55±1.174^b^ | 16.49±1.313^d^ | 11.63±0.6498^d^ | 4.487±0.7806^c^ |
| **Jejunum** | 1.006±0.02414 | 3.96±0.7072 | 7.593±1.725 | 2.789±0.4053^b^ | 1.771±0.3747^a^ | 1.247±0.3991 | 0.7243±0.1626 |
| **Ileum** | 1.032±0.04953 | 11.11±1.502^c^ | 28.74±3.148^c^ | 10.84±1.093 | 4.087±0.5481^c^ | 1.459±0.3384^a^ | 4.711±0.9012^b^ |
| **Colon** | 1.005±0.05892 | 2.456±0.3198 | 6.68±0.5936 | 8.493±1.131 | 0.6962±0.1552 | 0.5467±0.1162 | 0.4583±0.14 |
|  | **IL-10** | | | | | | |
|  | **ctrl** | **1h** | **2h** | **4h** | **8h** | **12h** | **24h** |
| **Duodenum** | 1.038±0.03733 | 6.415±0.3701^d^ | 2.355±0.3575^c^ | 26.36±1.769^d^ | 2.018±0.2379^b^ | 8.128±0.5683^d^ | 3.058±0.1927^d^ |
| **Jejunum** | 1.013±0.04733 | 7.243±1.03^c^ | 11.71±1.363^d^ | 3.605±0.7258 | 2.441±0.3622^b^ | 1.518±0.3464^a^ | 0.9187±0.2343 |
| **Ileum** | 0.9851±0.01922 | 1.068±0.3464 | 3.869±0.7089^b^ | 3.419±0.3212 | 1.287±0.2754 | 0.812±0.2398 | 0.9196±0.1852 |
| **Colon** | 1.024±0.02783 | 0.6003±0.1543 | 0.3174±0.1276 | 3.218±0.3244 | 0.6939±0.2918 | 0.5667±0.1231 | 0.5886±0.1914 |

Results expressed as Mean ± SEM. n = 6 mice in each group. The statistical analysis and comparison used the colon in each group as a control. ^a^P < 0.05, ^b^P < 0.01, ^c^P < 0.001, ^d^P < 0.0001 (Student's t-test).

**Figure S1: The mRNA expression level of lipocalin-2 in DSS-induced acute enteritis.**

**
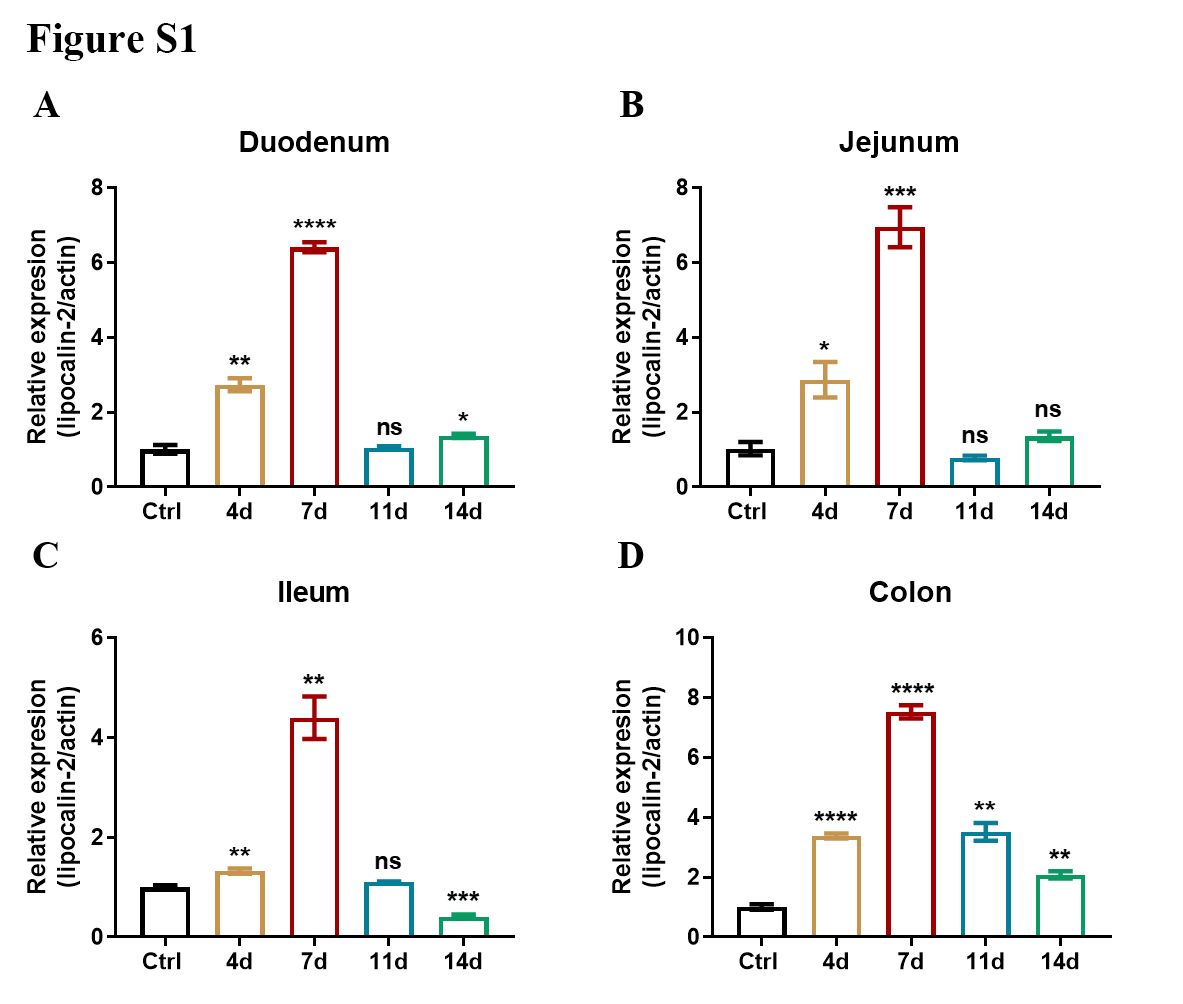
**

**Figure S1: The mRNA expression level of lipocalin-2 in DSS-induced acute enteritis.** (**A**) Deodenum. (**B**) Jejunum. (**C**) Ileum. (**D**) Colon. The data were represented as mean ± SEM. n = 3 mice in each group. **P* < 0.05, ***P* < 0.01, ****P* < 0.001, *****P* < 0.0001.

**Figure S2: Flow cytometric analysis of DSS-induced acute enteritis.**


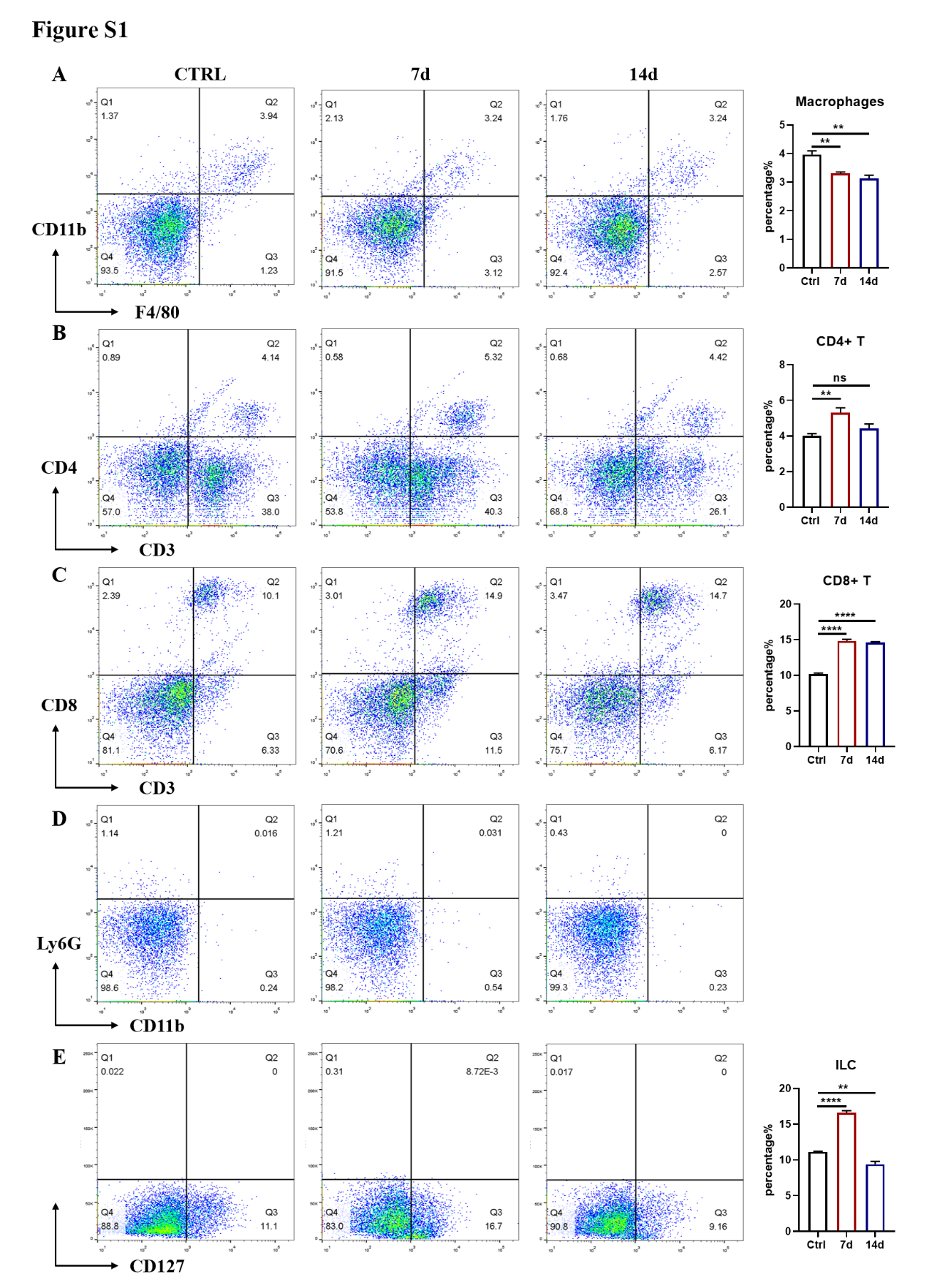


**Figure S2: Flow cytometric analysis of DSS-induced acute enteritis.** Scatter diagram of (**A**) macrophages, (**B**) CD4^+^ lymphocytes, (**C**) CD8^+^ lymphocytes, (**D**) neutrophils, and (**E**) CD127^+^ intrinsic lymphoid cells in the mouse intestine. **P* < 0.05, ***P* < 0.01, ****P* < 0.001, *****P* < 0.0001.

**Figure S3: The mRNA expression level of lipocalin-2 in anti-CD3 antibody-induced acute enteritis.**


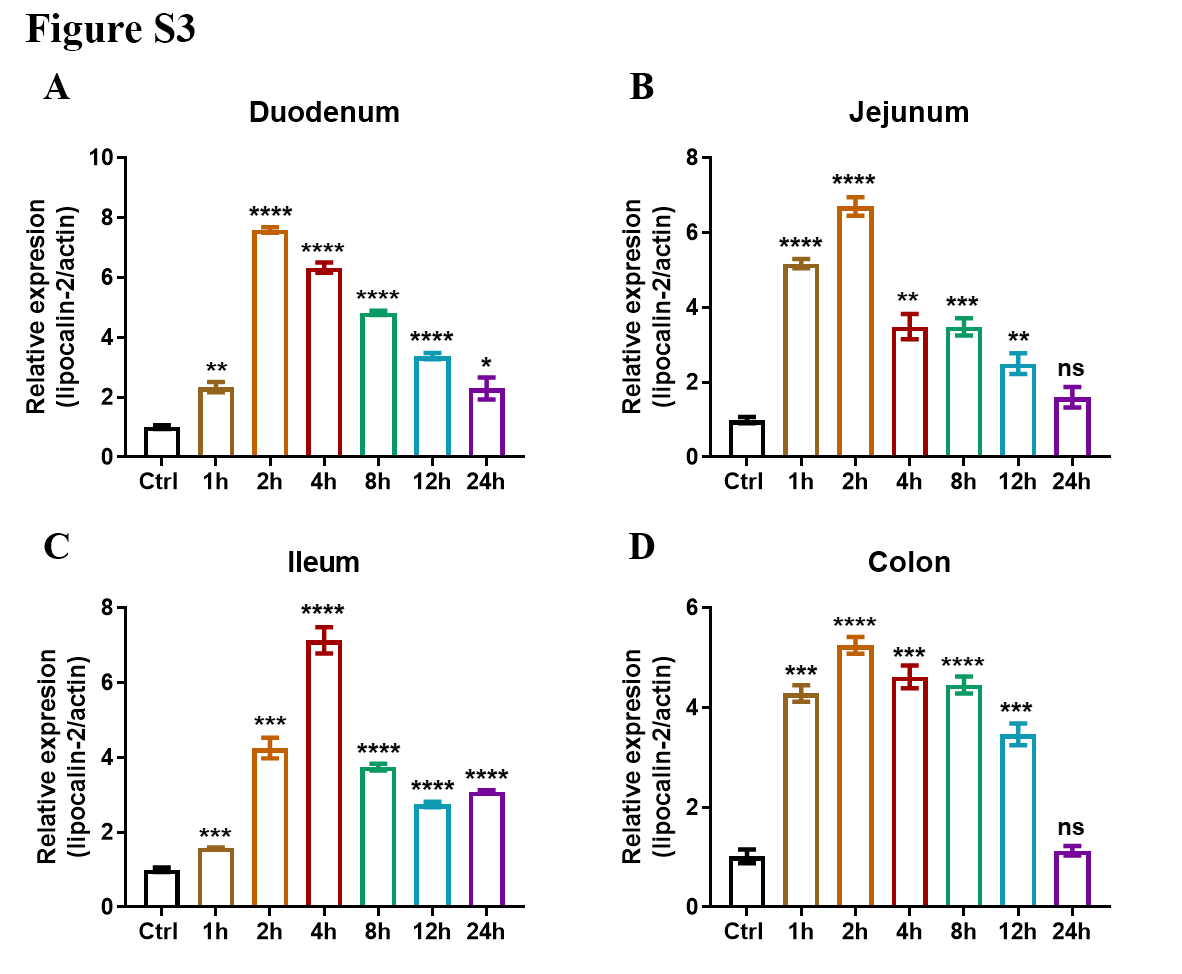


**Figure S3: The mRNA expression level of lipocalin-2 in anti-CD3 antibody-induced acute enteritis.** (**A**) Deodenum. (**B**) Jejunum. (**C**) Ileum. (**D**) Colon. The data were represented as mean ± SEM. n = 3 mice in each group. **P* < 0.05, ***P* < 0.01, ****P* < 0.001, *****P* < 0.0001.

**Figure S4: Flow cytometric analysis of anti-CD3 antibody-induced acute enteritis.**

**
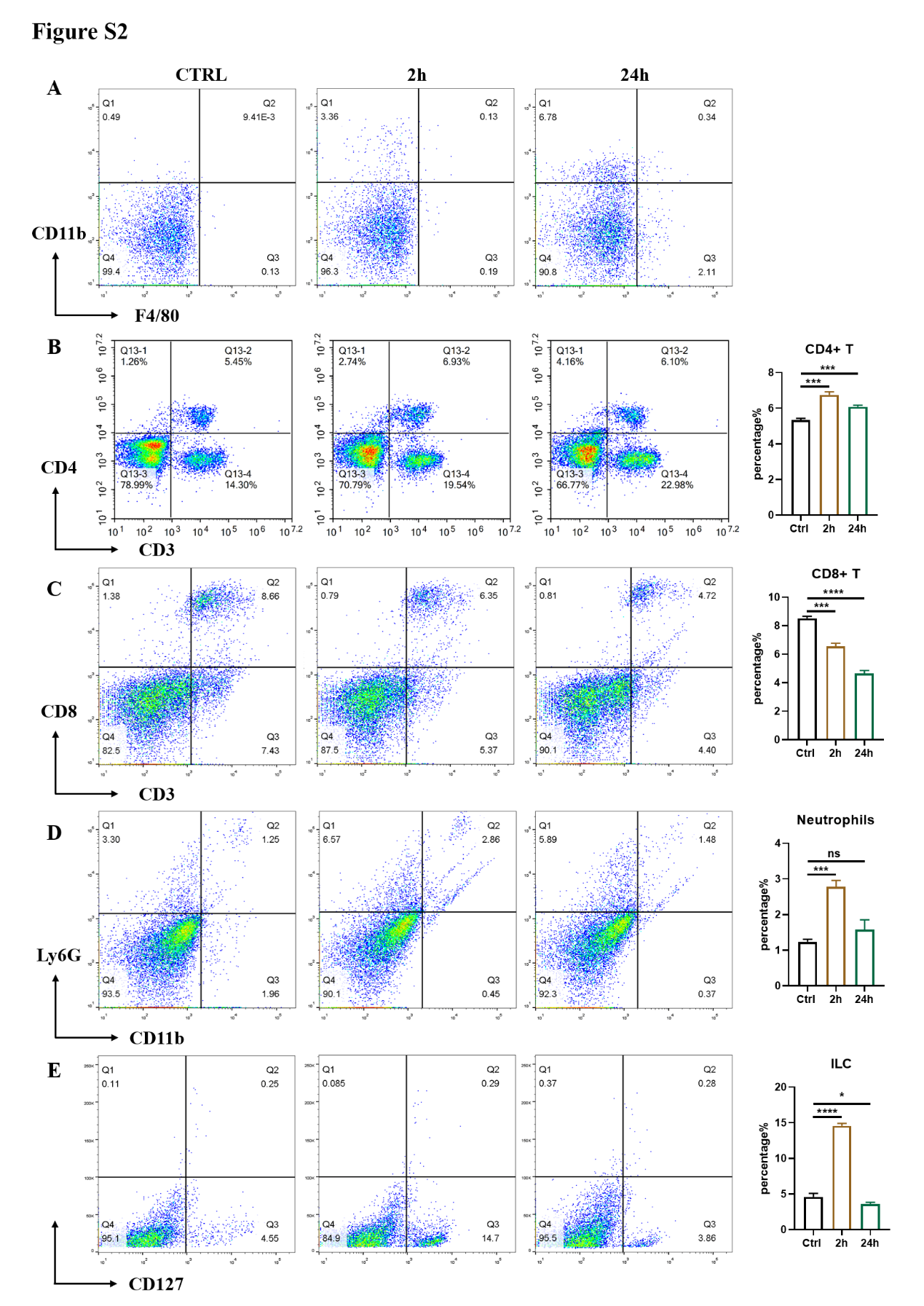
**

**Figure S4: Flow cytometric analysis of anti-CD3 antibody-induced acute enteritis.** Scatter diagram of (**A**) macrophages, (**B**) CD4^+^ lymphocytes, (**C**) CD8^+^ lymphocytes, (**D**) neutrophils, and (**E**) CD127^+^ intrinsic lymphoid cells in the mouse intestine. **P* < 0.05, ***P* < 0.01, ****P* < 0.001, *****P* < 0.0001.

**Figure S5: The mRNA expression level of lipocalin-2 in DSS-induced chronic enteritis.**

**
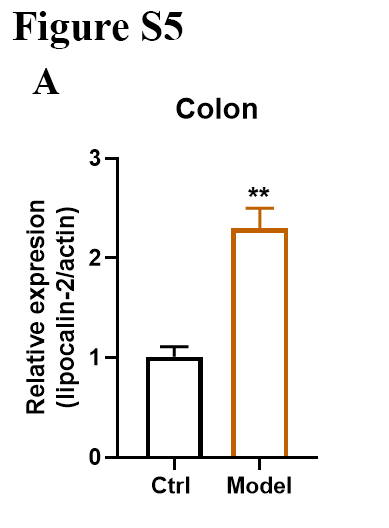
**

**Figure S5: The mRNA expression level of lipocalin-2 in DSS-induced chronic enteritis.** The data were represented as mean ± SEM. n = 3 mice in each group. **P* < 0.05, ***P* < 0.01, ****P* < 0.001, *****P* < 0.0001.

**Figure S6: Flow cytometric analysis of DSS-induced chronic enteritis.**

**
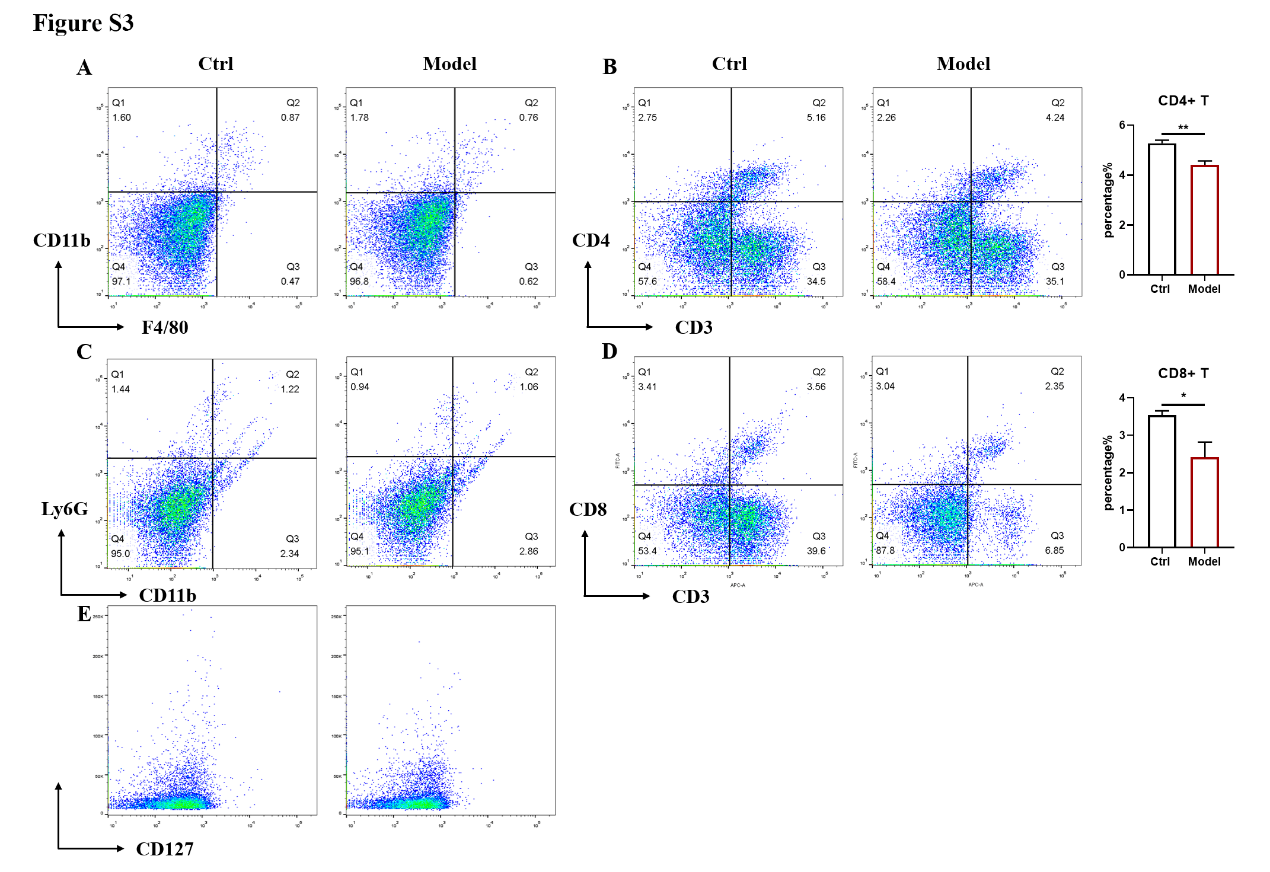
**

**Figure S6: Flow cytometric analysis of DSS-induced chronic enteritis.** Scatter diagram of (**A**) macrophages, (**B**) CD4^+^ lymphocytes, (**C**) neutrophils, (**D**) CD8^+^ lymphocytes and (**E**) CD127^+^ intrinsic lymphoid cells in the mouse intestine.

**Figure S7: Histological observation and inflammatory cytokines detection of duodenal, jejunal and ileal tissue in HFD-induced chronic enteritis.**


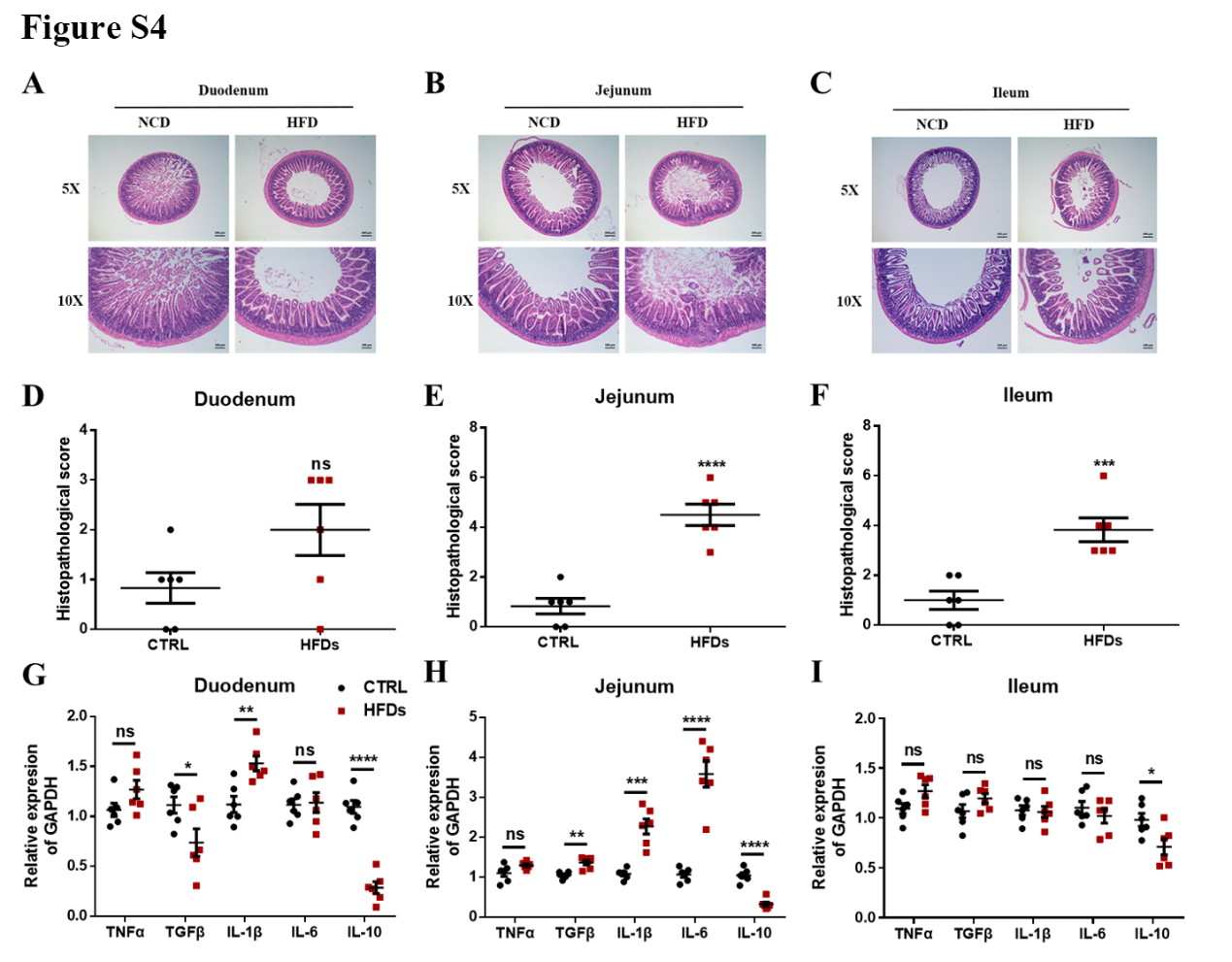


**Figure S7: Histological observation and inflammatory cytokines detection of duodenal, jejunal and ileal tissue in HFD-induced chronic enteritis.** (**A-C**) Representative paraffin sections of duodenal (A), jejunal (B) and ileal (C) tissues stained with H&E. Bars indicate 100 μm. (**D-F**) Histopathological scores of duodenal (D), jejunal (E) and ileal (F) tissue sections. (**G-I**) The mRNA expression levels of TNFα, TGFβ, IL-1β, IL-6 and IL-10 in duodenal (G), jejunal (H) and ileal (I) tissues were detected by quantitative real‐time PCR. Data were represented as mean ± SEM. n = 6 mice in each group. **P* < 0.05, ***P* < 0.01, ****P* < 0.001, *****P* < 0.0001.

**Figure S8: The mRNA expression level of lipocalin-2 in HFD-induced chronic enteritis.**

**
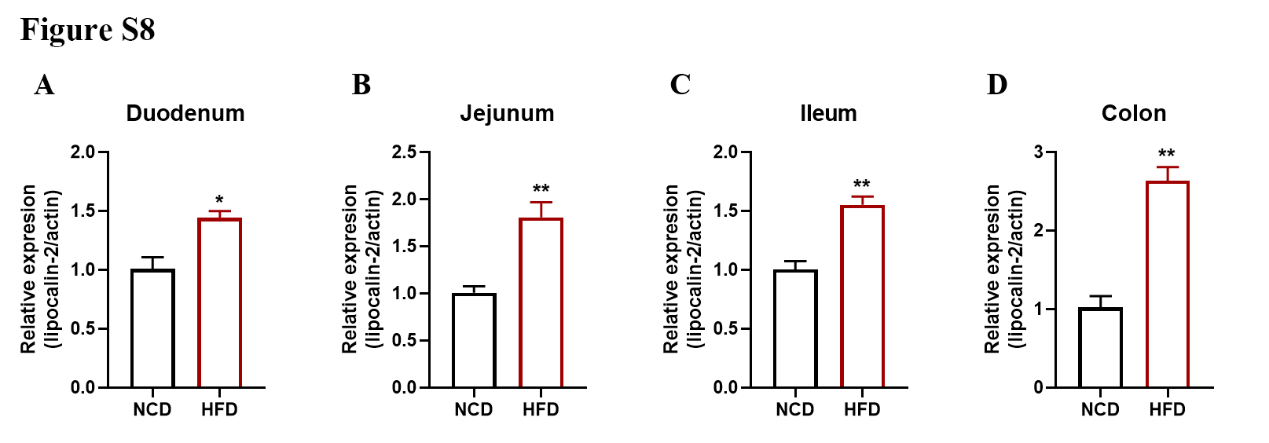
**

**Figure S8: The mRNA expression level of lipocalin-2 in HFD-induced chronic enteritis.** (**A**) Deodenum. (**B**) Jejunum. (**C**) Ileum. (**D**) Colon. The data were represented as mean ± SEM. n = 3 mice in each group. **P* < 0.05, ***P* < 0.01, ****P* < 0.001, *****P* < 0.0001.

**Figure S9: Flow cytometric analysis of HFD-induced chronic enteritis.**

**
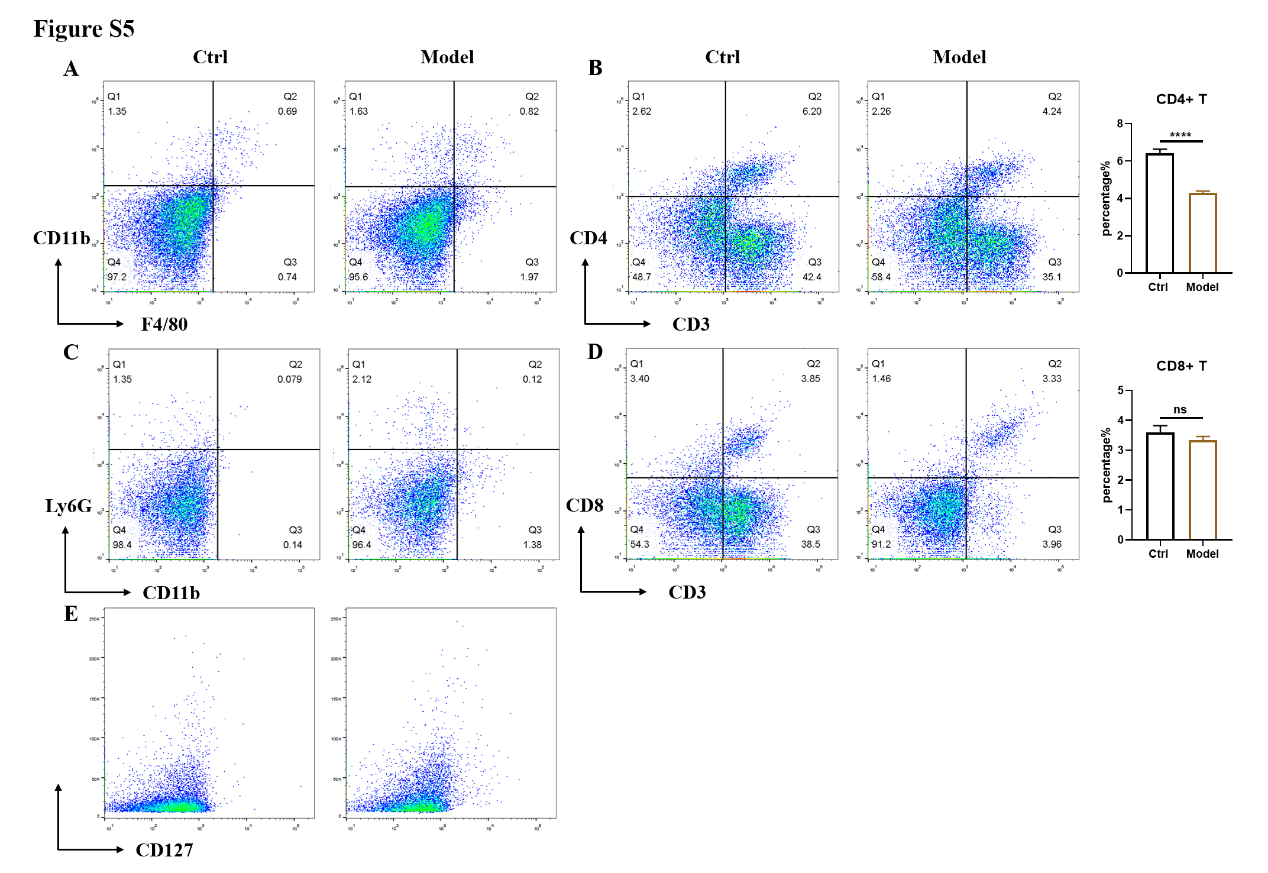
**

**Figure S9: Flow cytometric analysis of HFD-induced chronic enteritis.** Scatter diagram of (**A**) macrophages, (**B**) CD4^+^ lymphocytes, (**C**) neutrophils, (**D**) CD8^+^ lymphocytes and (**E**) CD127^+^ intrinsic lymphoid cells in the mouse intestine.

**Figure S10: Intestinal microbial detection of four enteritis models.**

**
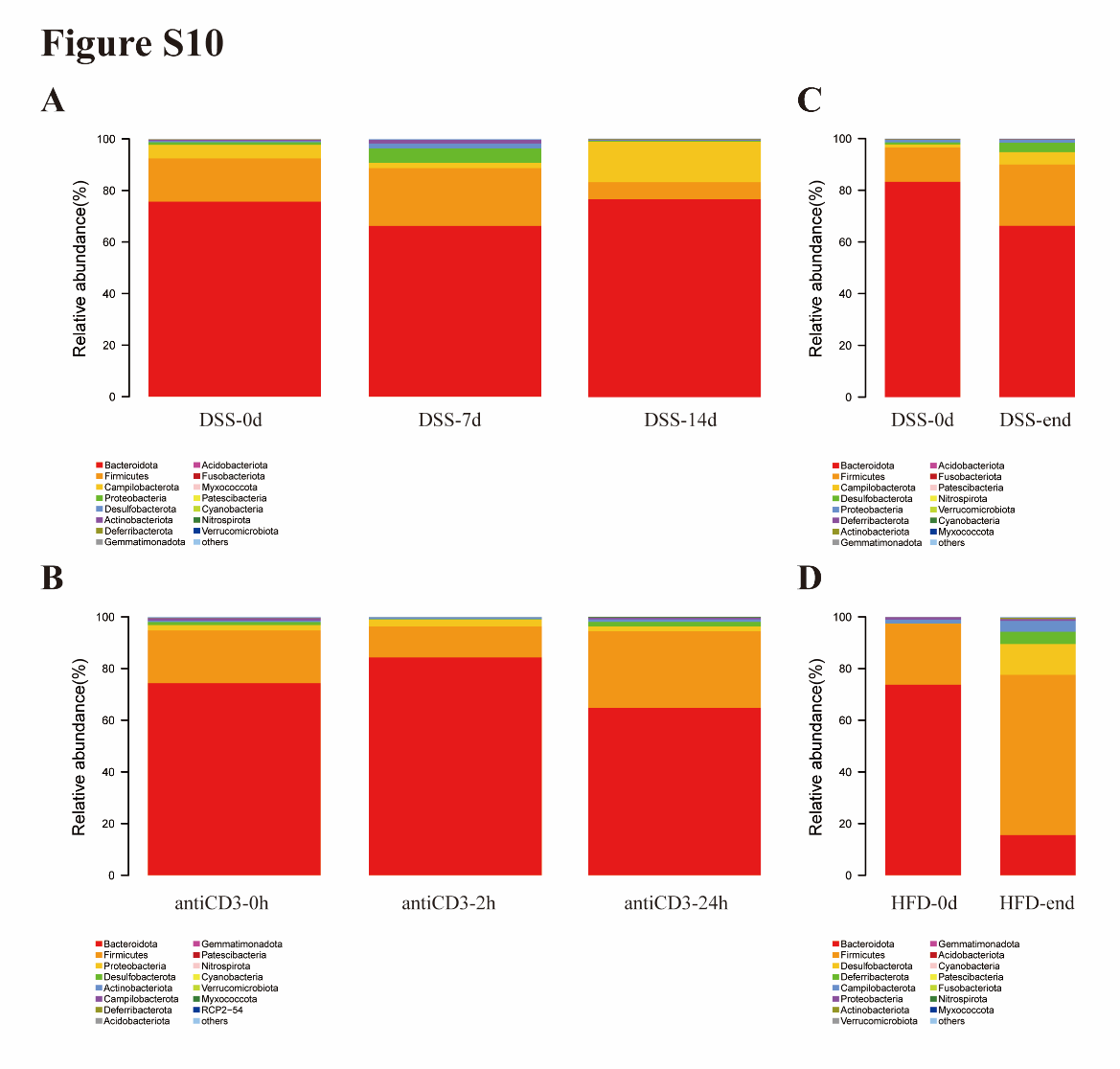
**

**Figure S10: Intestinal microbial detection of four enteritis models.** (**A**) In DSS-induced acute enteritis, the relative taxonomic abundance at the phylum level of gut microbiota. (**B**) In anri-CD3 antibody-induced hyperacute enteritis, the relative taxonomic abundance at the phylum level of gut microbiota. (**C**) In DSS-induced chronic enteritis, the relative taxonomic abundance at the phylum level of gut microbiota. (**D**) In HFD-induced chronic enteritis, the relative taxonomic abundance at the phylum level of gut microbiota.
